# Antidepressant drugs act by directly binding to TRKB neurotrophin receptors

**DOI:** 10.1016/j.cell.2021.01.034

**Published:** 2021-03-04

**Authors:** Plinio C. Casarotto, Mykhailo Girych, Senem M. Fred, Vera Kovaleva, Rafael Moliner, Giray Enkavi, Caroline Biojone, Cecilia Cannarozzo, Madhusmita Pryiadrashini Sahu, Katja Kaurinkoski, Cecilia A. Brunello, Anna Steinzeig, Frederike Winkel, Sudarshan Patil, Stefan Vestring, Tsvetan Serchov, Cassiano R.A.F. Diniz, Liina Laukkanen, Iseline Cardon, Hanna Antila, Tomasz Rog, Timo Petteri Piepponen, Clive R. Bramham, Claus Normann, Sari E. Lauri, Mart Saarma, Ilpo Vattulainen, Eero Castrén

**Affiliations:** 1 Neuroscience Center-HILIFE, University of Helsinki, Helsinki, Finland; 2 Department of Physics, University of Helsinki, Helsinki, Finland; 3 Institute of Biotechnology-HILIFE, University of Helsinki, Helsinki, Finland; 4 Department of Biomedicine and KG Jebsen Center for Research on Neuropsychiatric Disorders, University of Bergen, Bergen, Norway; 5 Department of Psychiatry and Psychotherapy, Medical Center-University of Freiburg, Faculty of Medicine, University of Freiburg, Freiburg, Germany; 6 Berta-Ottenstein-Programme for Clinician Scientists, Faculty of Medicine, University of Freiburg, Freiburg, Germany; 7 Department of Pharmacology, Ribeirão Preto Medical School, University of São Paulo, São Paul, Brazil; 8 Brain Master Program, Faculty of Science, Aix-Marseille Université, Marseille, France; 9 Department of Psychiatry, University of Regensburg, Regenburg, Germany; 10 Department of Neuroscience, Perelman School of Medicine, University of Pennsylvania, Philadelphia, PA, USA; 11 Division of Pharmacology and Pharmacotherapy, Faculty of Pharmacy, University of Helsinki, Helsinki, Finland; 12 Center for Basics in Neuromodulation (NeuroModul Basics), University of Freiburg, Freiburg, Germany; 13 Molecular and Integrative Biosciences Research Program, University of Helsinki, Helsinki, Finland; 14 Computational Physics Laboratory, Tampere University, Tampere, Finland

**Keywords:** antidepressant, neurotrophin, BDNF, cholesterol, plasticity, fluoxetine, ketamine, molecular dynamic simulation

## Abstract

It is unclear how binding of antidepressant drugs to their targets gives rise to the clinical antidepressant effect. We discovered that the transmembrane domain of tyrosine kinase receptor 2 (TRKB), the brain-derived neurotrophic factor (BDNF) receptor that promotes neuronal plasticity and antidepressant responses, has a cholesterol-sensing function that mediates synaptic effects of cholesterol. We then found that both typical and fast-acting antidepressants directly bind to TRKB, thereby facilitating synaptic localization of TRKB and its activation by BDNF. Extensive computational approaches including atomistic molecular dynamics simulations revealed a binding site at the transmembrane region of TRKB dimers. Mutation of the TRKB antidepressant-binding motif impaired cellular, behavioral, and plasticity-promoting responses to antidepressants *in vitro* and *in vivo*. We suggest that binding to TRKB and allosteric facilitation of BDNF signaling is the common mechanism for antidepressant action, which may explain why typical antidepressants act slowly and how molecular effects of antidepressants are translated into clinical mood recovery.

## Highlights

• Several antidepressants, including SSRIs and ketamine, directly bind to TRKB
• TRKB dimerization at transmembrane region forms a binding pocket for fluoxetine
• Antidepressant binding to TRKB facilitates BDNF action and plasticity
• Point mutation in TRKB transmembrane region blocks the effects of antidepressants

## Graphical Abstract

**Figure:**
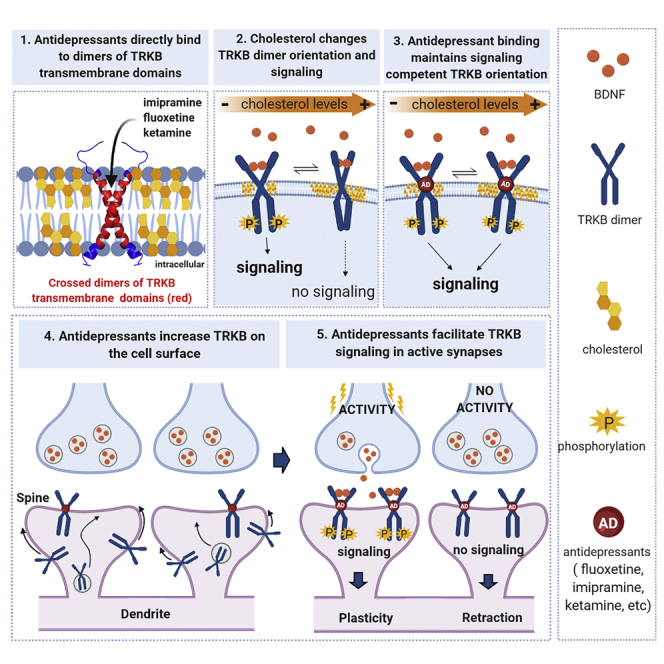

## Teaser

Direct binding of both typical and fast-acting antidepressants to the BDNF receptor TRKB accounts for cell biological and behavioral actions of antidepressants. This mechanism directly connects antidepressant action to neuronal plasticity and may explain the slow action of typical antidepressants.

## Introduction

Several targets for antidepressant (AD) drug action have been identified, but it is not clear how binding to these targets translates into clinical effects. Typical ADs such as tricyclic ADs (TCA), serotonin selective reuptake inhibitors (SSRI), and monoamine oxidase inhibitors (MAOI), increase the synaptic levels of monoamines by inhibiting their reuptake or metabolism, but it is unclear why their clinical effects are delayed, while the effects on monoamines are fast (Belmaker and Agam, 2008; Malhi and Mann, 2018). The rapid AD effect of ketamine (KET) is attributed to inhibition of NMDA-type glutamate receptors (Abdallah et al., 2015; Berman et al., 2000; Zarate et al., 2006). However, 2R,6R-hydroxynorketamine (R,R-HNK), a KET metabolite with AD-like activity, exhibits low affinity to NMDA receptors, which has called the role of NMDA receptors in the KET action into question (Zanos et al., 2016, 2018).

Essentially all ADs, including KET and R,R-HNK, increase the expression and signaling of brain-derived neurotrophic factor (BDNF) through neurotrophic tyrosine kinase receptor 2 (TRKB) (Autry and Monteggia, 2012; Castrén and Antila, 2017; Duman and Monteggia, 2006). The effects of SSRIs and KET on BDNF signaling have been considered to be indirect, through the inhibition of serotonin transporter (5HTT) and NMDA receptors, respectively. BDNF mimics the effects of ADs in rodents and inhibiting TRKB signaling prevents their behavioral effects (Duman and Monteggia, 2006; Saarelainen et al., 2003). Activation of TRKB is a critical mediator of activity-dependent synaptic plasticity (Park and Poo, 2013), and the AD-induced TRKB signaling reactivates a state of juvenile-like plasticity in the adult brain, which has been suggested to underlie the effects of ADs on mood (Castrén and Antila, 2017; Karpova et al., 2011; Maya Vetencourt et al., 2008).

TRKB signaling is bidirectionally linked to brain cholesterol (CHOL) metabolism. BDNF promotes production of CHOL in neurons (Suzuki et al., 2007; Zonta and Minichiello, 2013) and CHOL regulates TRKB signaling (Pereira and Chao, 2007; Suzuki et al., 2004). CHOL is essential for neuronal maturation and proper synaptic transmission (Martin et al., 2014; Mauch et al., 2001), but it does not pass the blood-brain barrier, therefore, neurons are dependent on CHOL synthesized by astrocytes and transported through an ApoE-mediated mechanism (Pfrieger and Ungerer, 2011). Synaptic CHOL levels are low during the embryonic and early postnatal life but strongly increase during the 3^rd^ postnatal week in mice (Suzuki et al., 2004; Tulodziecka et al., 2016), which coincides with the increase in BDNF expression and appearance of ADs effects on TRKB (Di Lieto et al., 2012). Many ADs interact with phospholipids and accumulate in CHOL-rich membrane domains, such as lipid rafts (Erb et al., 2016; Wray et al., 2019).

These data prompted us to investigate the potential interactions between TRKB, CHOL, and ADs. We found that the TRKB transmembrane domain (TMD) senses changes in the cell membrane CHOL levels, and we elucidated its mechanism. Furthermore, we found that different AD drugs directly bind to a site formed by a dimer of TRKB TMDs, thereby facilitating cell surface expression of TRKB and promoting BDNF signaling. These data suggest that direct binding to TRKB and promotion of BDNF-mediated plasticity is a mechanism of action for AD drugs.

## Results

### Cholesterol sensing by TRKB

**Figure 1:**
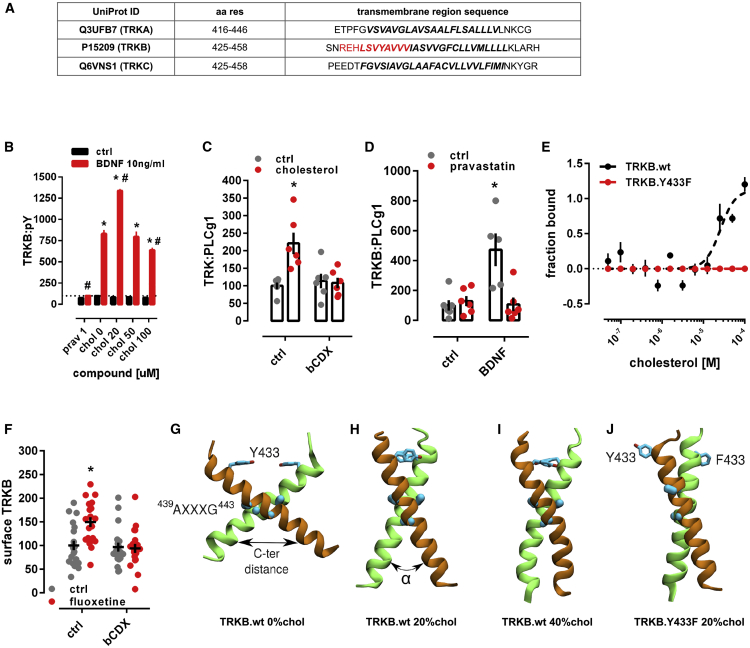
Cholesterol sensing by TRKB (A) Identification of CARC motif (red) in the TM domain of TRKB, but not TRKA or TRKC. (B) Cholesterol promotes the effects of BDNF on TRKB autophosphorylation (TRKB:pY) at moderate, but inhibits BDNF at low or high concentrations (interaction: F[5,84] = 5.654, p = 0.0002; n = 6/group). Cultured cortical cells received cholesterol (15 min) followed by BDNF or cholesterol (15 min) and were submitted to ELISA for TRKB:pY. (C) β-cyclodextrin (bCDX, 2 mM, 30 min) prevents BDNF-induced increase in TRKB-PLC-γ1 interaction (TRK:PLCg1) (interaction: F[1,20] = 9.608, p = 0.0056, n = 6/group). (D) Pravastatin (1 μM, 3 days) also blocks the BDNF-induced increase in TRKB:PLC-γ1 interaction (interaction: F[1,19] = 11.23, p = 0.003; n = 5–6). ^∗^p < 0.05 from the ctrl/ctrl group, #p < 0.05 from ctrl/chol0 group, data expressed as mean ± SEM of percentage from control group. (E) Microscale thermophoresis demonstrated direct interaction between GFP-tagged TRKB and cholesterol (15 min) in lysates from GFP-TRKB expressing HEK293T cells; mutation of Y433F blocks this interaction in MST (interaction: F[11,72] = 15.25, p < 0.0001, n = 4). (F) Fluoxetine-induced increase in TRKB surface exposure is blocked by bCDX (interaction: F[1,73] = 7.022, p = 0.0099, n = 19–20). (G–J) Structure of wild-type TRKB (G) in the absence of cholesterol and (H) at cholesterol concentrations of 20 mol% and (I) 40 mol%, and (J) for the heterodimer of TRKB.wt and TRKB.Y433F at 20 mol %. Related to systems 5–8 in Table S1 and Figure S2 for distance and α values between C termini. See also Figures S1, S2, and S3.

**Figure S1:**
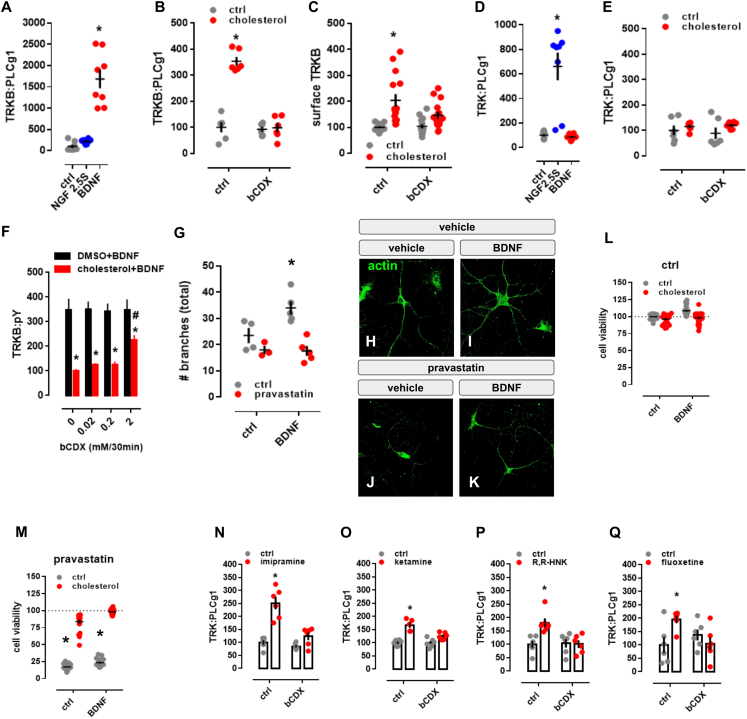
Cholesterol sensing by TRKB, related to Figure 1 (A-E) MG87 cells were treated with β-cyclodextrin (bCDX), NGF, BDNF, or cholesterol and the levels of TRKB:PLC-γ1 or surface TRKB determined by ELISA. In MG87 cells expressing TRKB, (A) BDNF (10ng/ml/15min), but not NGF (50ng/ml/15min), increases the TRKB:PLC-γ1 interaction [treatment: F(2,21) = 46.24; p = 0.0001] measured by ELISA. The effect of cholesterol (20μM/15min) on (B) TRKB:PLC-γ1 coupling [interaction: F(1,20) = 59.49; p = 0.0001] and (C) surface positioning of TRKB [interaction: F(1,54) = 4.202; p = 0.04] is counteracted by pre-treatment with beta-cyclodextrin (bCDX; 2mM/30min). In MG87 cells expressing TRKA, (D) NGF, but not, BDNF, increases the TRKA:PLC-γ1 coupling [treatment: F(2,21) = 25.29; p = 0.0001]. (E) Lack of effect of cholesterol-induced TRKA:PLC-γ1 in cells expressing TRKA [interaction: F(1,20) = 0.25; p = 0.64]. (F) Rat cortical cells were treated with different concentrations of bCDX (30min), challenged by a combo of cholesterol+BDNF (15min), and the levels of TRKB:pY was determined by ELISA. β-cyclodextrin (mM/30min) reverses the block of BDNF-induced pTRKB (10ng/ml/15min) by high cholesterol concentration (100 μM/15min) [F(1,40) = 96.95, p < 0.0001, n = 6/group]. (G) Rat hippocampal cells were treated with pravastatin and BDNF, fixed and stained for actin. Effect of pravastatin (1μM/3days) on BDNF-induced neurite branching (10ng/ml/3days); interaction: F(1,13) = 4.967, p = 0.0441, n = 3-5]. (H-K) representative images of pravastatin effect on BDNF-induced branching. (L,M) Rat cortical cells were treated with pravastatin, cholesterol and BDNF, and the cell viability determined by CellTiterGlo. Pravastatin-induced cell death (2 μM/5days) is counteracted by co-incubation with cholesterol (20μM/5days) and BDNF (10ng/ml/5days) [interaction: F(1,164) = 10.895, p = 0.001, n = 20-24]. Data expressed as mean ± SEM of percentage from ctrl group. ^∗^p < 0.05 from the control group (Fisher’s LSD). (N-Q) Rat cortical cells were treated with β-cyclodextrin (bCDX) or antidepressants, and the levels of TRKB:PLC-γ1 determined by ELISA. The pretreatment with bCDX (2mM/30min) prevents the increase in TRKB:PLC-γ1 (PLCg1) induced by (N) imipramine [interaction: F(1,20) = 14.71, p = 0.0010, n = 6/group], (O) ketamine [interaction: F(1,19) = 9.335, p = 0.0065, n = 5-6], (P) R,R-HNK [interaction: F(1,20) = 8.033, p = 0.0102, n = 6/group] or (Q) fluoxetine [interaction: F(1,20) = 8.035, p = 0.0103, n = 6/group]. ^∗^p < 0.05 from the control group (Fisher’s LSD).

CHOL is known to promote neuronal maturation and plasticity, but how it exerts these effects is unclear (Mauch et al., 2001; Pfrieger and Ungerer, 2011). CHOL is proposed to interact with proteins through the so-called CHOL-recognition and alignment consensus (CRAC) domain or its inverted version CARC (Fantini et al., 2019). We identified a CARC motif in the TRKB transmembrane (TM) region. This sequence is specific to TRKB and is not present in other TRK receptors (Figure 1A), suggesting that CHOL might directly interact with TRKB. Indeed, addition of CHOL at 20 μM to the culture media enhanced TRKB phosphorylation (pTRKB) by BDNF (10 ng/mL) in primary cortical neurons (Figure 1B). However, at higher concentrations (50–100 μM), CHOL suppressed the effects of BDNF (Figure 1B). CHOL promoted the interaction of TRKB, but not of TRKA, with phospholipase C-γ1 (PLC-γ1) (Figures S1A–S1E), a critical mediator of TRKB intracellular signaling (Minichiello et al., 2002), and this effect was blocked by beta-cyclodextrin (βCDX), a CHOL-sequestering agent, at a dose that counteracts CHOL effects (Figures 1C and S1F). Microscale thermophoresis (MST) (Jerabek-Willemsen et al., 2014) experiments demonstrated that CHOL (10–100 μM) directly interacts with GFP-TRKB in HEK293T cell lysates with an affinity of ∼20 μM (Figure 1E).

TRKB mostly resides in intracellular vesicles not accessible to BDNF (Du et al., 2000; Haapasalo et al., 2002; Meyer-Franke et al., 1998). We found that CHOL treatment increased cell surface translocation of TRKB (Figure S1C). The effects of BDNF on TRKB-PLC-γ1 interaction (Figure 1D) and on the neurite branching in cultured neurons (Figures S1G–S1K) were prevented by a CHOL synthesis inhibitor pravastatin (1 μM/3 days), as reported previously (Suzuki et al., 2004). At 2 μM for 5 days, pravastatin reduced neuronal survival that was rescued by CHOL (20 μM), but not by BDNF (Figures S1L and S1M).

**Figure S2:**
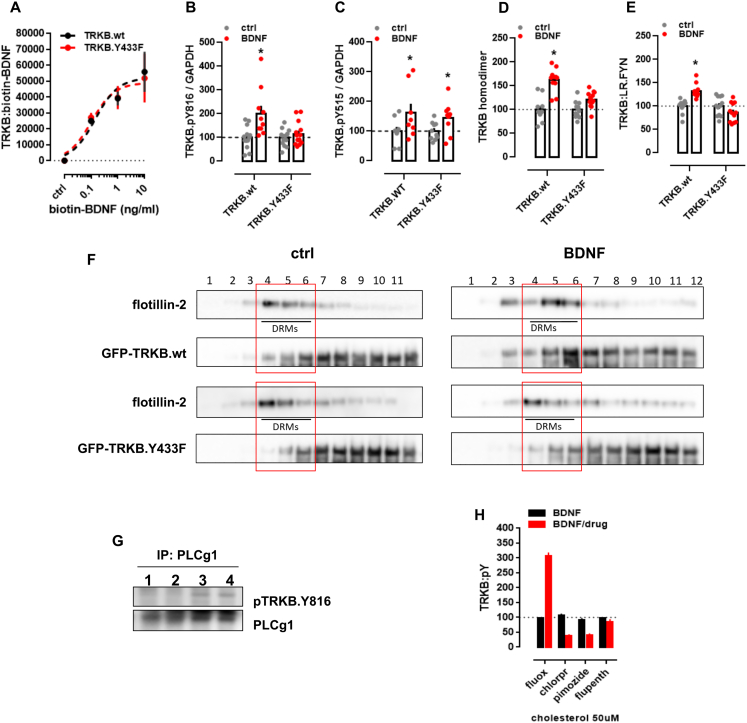
Antidepressants bind to TRKB transmembrane domain, related to Figure 2 (A) Lysates from HEK293T cells transfected to express TRKB were submitted to ligand binding assay. BDNF interaction with TRKB is not altered by the Y433F mutation (n = 6/group). See schematics in S5A. (B,C) MG87 cells transfected to express TRKB were treated with BDNF and the levels of pTRKB determined by western-blotting. BDNF-induced phosphorylation of TRKB at (B) Y816 is prevented in the TRKB.Y433F mutant [interaction: F(1,47) = 6.688, p = 0.0129; n = 10-14], but the Y433F mutation does not affect BDNF-induced phosphorylation of TRKB at (C) Y515 residues in MG87 cells [interaction: F(1,33) = 0.1874, p = 0.6679; n = 9-10]. (D,E) N2A cells transfected to express luciferase-tagged TRKB and/or raft-restricted FYN, were treated with BDNF and submitted to PCA. (D) The BDNF-induced dimerization of TRKB is compromised by the Y433F mutation [interaction: F(1,42) = 11.08, p = 0.0018; n = 11-12]. (E) The BDNF-induced increase in TRKB interaction with FYN fragment in lipid raft is compromised by the Y433F mutation [interaction: F(1,44) = 20.96, p < 0.000; n = 12]. Data expressed as mean ± SEM of percentage from ctrl group. ^∗^p < 0.05 from the control group (Fisher’s LSD). (F) N2A cells transfected to express TRKB were treated with BDNF and submitted to fractionation of membrane components. The Y433F mutation prevents BDNF-induced (10ng/ml/15min) translocation of TRKB to lipid-rafts in N2A cells (DRM: detergent-resistant membranes; 1 of 2 replicas). Rat cortical cells were treated with bCDX and fluoxetine, and the levels of surface TRKB determined by ELISA. Rat cortical cells were treated with fluoxetine or ketamine and submitted to immunoprecipitation of PLC-γ1 and western-blotting for TRKB and PLC-γ1. (G) Representative western-blotting of co-immunoprecipitation of PLC-γ1 and TRKB phosphorylated at Y816 in cultured cortical cells of rat embryo (1 of 2 replicas); lane 1: ctrl, 2: ctrl, 3: fluoxetine (10 μM/15min), 4: ketamine (10 μM/15min). (H) Rat cortical cells were preincubated with cholesterol (50uM) and fluoxetine, chlorpromazine, pimozide or flupenthixol (10uM) for 15min and challenged with BDNF (10ng/ml/15min). The levels of TRKB:pY were determined by ELISA [interaction: F(3,64) = 181.9, p < 0.0001, n = 9/group]. Data expressed as mean ± SEM of percentage from ctrl group. ^∗^p < 0.05 from the control group (Fisher’s LSD). ^∗^p < 0.05 from the control group (Fisher’s LSD).

Mutation of TRKB tyrosine 433, a predicted key residue in the CARC motif (Fantini and Barrantes, 2013) to phenylalanine (TRKB.Y433F), did not influence the binding affinity of BDNF (TRKB.wt = 3.1 pM; TRKB.Y433F = 2.9 pM) (Figure S2A), but it compromised CHOL sensing of TRKB (Figure 1E) and reduced the BDNF-induced increase in the phosphorylation of TRKB at the PLC-γ1 interaction site Y816, but not at Y515 (Figures S2B and S2C). Split luciferase protein complementation assay (Merezhko et al., 2020) indicated that although Y433F mutation did not influence the basal TRKB dimerization, it compromised BDNF-induced increase in TRKB dimerization (Figure S2D). Furthermore, BDNF-induced translocation of TRKB.Y433F to lipid rafts (Figure S2F) and its interaction with the raft-restricted FYN (Pereira and Chao, 2007) was reduced when compared to the wild-type TRKB (Figure S2E). These data indicate that the Y433 in the TRKB CARC domain is important for BDNF-induced translocation of TRKB to lipid-raft regions on the neuronal surface, thereby promoting BDNF signaling.

### Modeling of cholesterol-TRKB interaction

**Figure S3:**
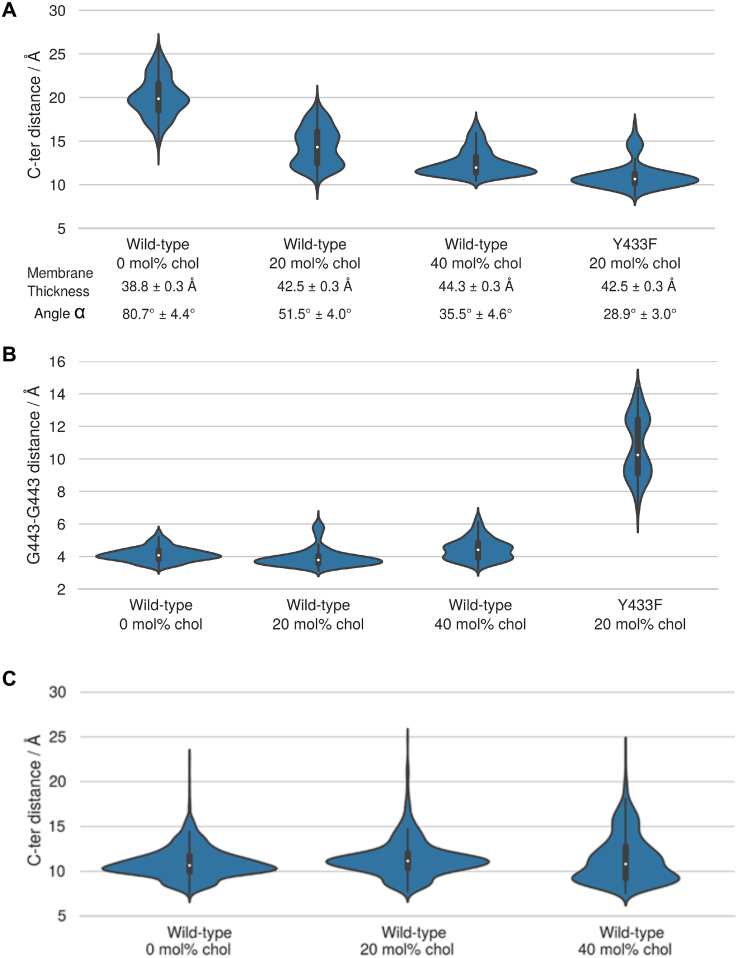
Cholesterol sensing by TRKB, related to Figures 1 and 3 (A) The distribution of the distance between the C-terminal residues of the monomers (center of mass L451-L453 Cα atoms (indicated with an arrow in Figure 1) are shown as violin plots. Increasing cholesterol concentration increases membrane thickness, which for the wild-type decreases the C-terminal distance. Y433F results in the disruption of the dimerization interface and the cross-like conformation. The parallel-like conformation of the WT-Y433F dimer appears to have a smaller hydrophobic length than that of the individual WT helices. Given at the bottom are average values for the membrane thickness (phosphate-phosphate distance) and the average angle between the helices ɑ. [Kruskal-Wallis: H = 27.8736; p < 0.001; n = 10/group]. (B) The effect of cholesterol concentration and the Y433F mutation on the stability of the interdimeric interface. The stability of the dimerization interface is characterized by a distribution of the distance between the monomers’ Cα carbons of G443 shown as violin plots for wild-type at different cholesterol concentrations and for the Y433F heterozygous mutant at 20 mol% cholesterol concentration (systems 1-4, Table S1) [Kruskal-Wallis: H = 25.4385; p < 0.001; n = 10/group]. The results demonstrate that the Y433F mutation results in a total disruption of the A439-G443 dimerization interface. (C) The distribution of the distance between the C-terminal residues of the monomers (center of mass L439-L437 Cα atoms) in the TRKA transmembrane domain shown as violin plots. The results indicate that cholesterol concentration has no notable effect on the distance between the C-terminal residues of the two monomers in the TRKA TM dimer. In essence, TRKA is non-responsive to changes in cholesterol concentration (systems 12-14, Table S1).

We next used atomistic molecular dynamics (MD) simulations to investigate the organization of TRKB TMD dimers (Table S1). Using a docking algorithm, we modeled five TMD dimer structures to initiate MD simulations, which showed that only one of them is stable in a phosphatidylcholine bilayer with 20–40 mol % CHOL. The stable structure features a cross-like conformation, where the two TMD helices interact at ^439^AXXXG^443^ (Figures 1G–1I), a GXXXG-like dimerization motif (Figure 1G) (Li et al., 2012; Senes et al., 2004). A similar cross-like conformation was proposed for the EGF receptor, where the distance between the C-termini of TMDs determines EGF signaling (Arkhipov et al., 2013; Endres et al., 2013; Sinclair et al., 2018). The average distance between the C termini of TRKB TMDs at CHOL concentrations of 0, 20, and 40 mol% was 19.4 Å, 14.3 Å, and 12.4 Å, respectively (Figure S3A).

Additional simulations revealed that the conformation of TRKB TMD dimers is sensitive to CHOL. As the fixed-length hydrophobic TMD helices reduce their tilt to match the thicker membrane at higher CHOL concentration, the stable cross-like dimer conformation seen at 20 mol% CHOL switched to a more parallel conformation at 40 mol% (Figures 1H and 1I). The Y433F mutation induced a 40° rotation of the TMD helices relative to each other (Figure 1J). This compromises the contact at the ^439^AXXXG^443^ motif and reduces the C-terminal distance of the TMDs (Figures S3A and S3B). In TRKA, which has no CARC or GXXXG-like domains, different CHOL concentrations did not influence the TMD dimer conformation (Figure S3C). These findings are consistent with our experimental data showing an optimal CHOL concentration for TKRB function (Figure 1B), which is compromised as the TMD helices are separated at the C terminus at low CHOL concentration and adopt an unstable parallel TM orientation at high CHOL concentration (Figure S3A).

### Antidepressants bind to TRKB transmembrane domain

**Figure 2:**
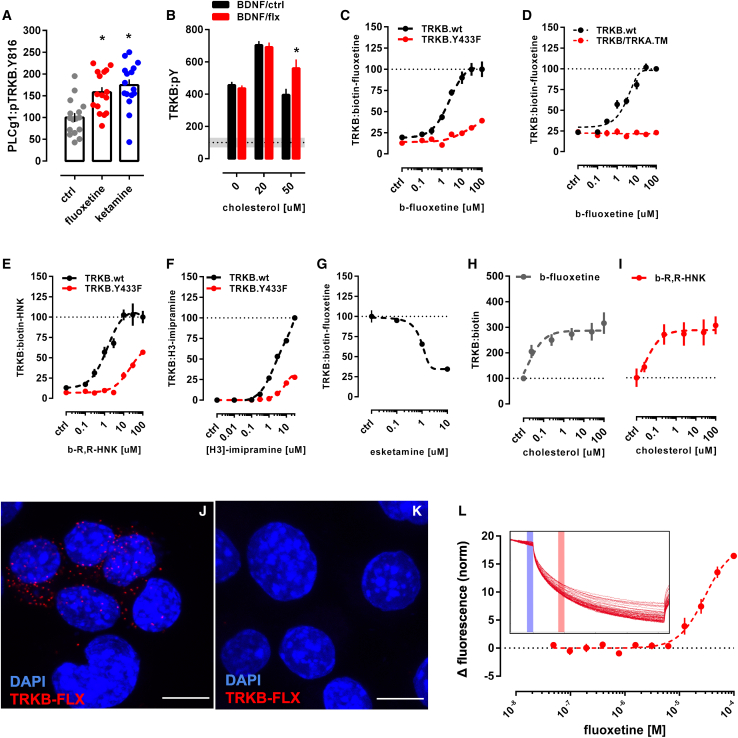
Antidepressants bind to TRKB transmembrane domain (A) Fluoxetine (10 μM/15 min) and ketamine (10 μM/15 min) increased pTRKB.Y816 in cortical neurons immunoprecipitated with anti-PLC-γ1 (F[2,45] = 11.03, p = 0.0001, n = 16/group). (B) Fluoxetine facilitates BDNF-induced activation of TRKB under high cholesterol concentrations (interaction: F[2,132] = 5.15, p = 0.0070, n = 12/group) in cultured cortical cells. (C and D) Biotinylated fluoxetine binds to TRKB in lysates of TRKB expressing HEK cells (interaction: F[7,153] = 16.18, p < 0.0001; n = 6–14), but not (C) to TRKB.Y433F mutant or (D) to TRKB carrying the TMD of TRKA (TRKB/TRKA.TM) (interaction: F[7,80] = 43.75, p < 0.0001, n = 6/group). (E and F) Binding of biotinylated R,R-HNK (interaction: F[7,160] = 14.91, p < 0.0001; n = 6–14) (E) and tritiated imipramine (interaction: F[7,16] = 106.1, p < 0.0001; n = 2) (F) to TRKB, but not to TRKB.Y433F. Data expressed mean ± SEM of percentage of binding at 100 μM for fluoxetine and R,R-HNK or at 30 μM for imipramine. (G) Esketamine displaces the interaction of biotinylated fluoxetine (1 μM) with TRKB (n = 8/group). (H and I) Cholesterol facilitates the interaction of (H) biotinylated fluoxetine (F[5,30] = 7.198, p = 0.0002, n = 6/group)and (I) R,R-HNK (F[5,30] = 4.592, p = 0.0031, n = 6/group) with TRKB. (J) *In situ* PLA demonstrates close proximity between biotinylated fluoxetine and TRKB on TRKB-expressing N2A cells (red dots). (K) No PLA signal is seen in cells not expressing TRKB. Blue, DAPI; scale bar, 10 μm. (L) MST demonstrated direct interaction between fluoxetine and GFP-tagged TRKB (15 min) in lysates from GFP-TRKB expressing HEK293T cells (n = 4/group). Experimental traces depicted in the inset, vertical bars: blue, fluorescence cold; red, fluorescence hot. See also Figures S2, S4, and S5.

Essentially all ADs promote TRKB signaling in rodents and this signaling is required for their behavioral effects (Castrén and Antila, 2017; Monteggia et al., 2004; Saarelainen et al., 2003). Many AD drugs are cationic amphipathic molecules that interact with phospholipids and accumulate at the lipid rafts (Chen et al., 2012; Erb et al., 2016; Kornhuber et al., 1995; Wray et al., 2019). We found that fluoxetine (FLX) and KET enhanced pTRKB at Y816 (Figure 2A) and FLX increased the surface expression of TRKB in primary cortical neurons (Figure 1F). FLX, imipramine, KET, and R,R-HNK increased TRKB interaction with PLC-γ1 and their effects were blocked by βCDX (Figures 1C and S1N–S1Q), which indicates that CHOL modulates AD-induced TRKB signaling. FLX partially rescued the reduction in BDNF-induced pTRKB response observed under high-CHOL (Figure 2B), suggesting that ADs promote TRKB signaling particularly in synaptic-like membranes rich in CHOL.

**Figure S4:**
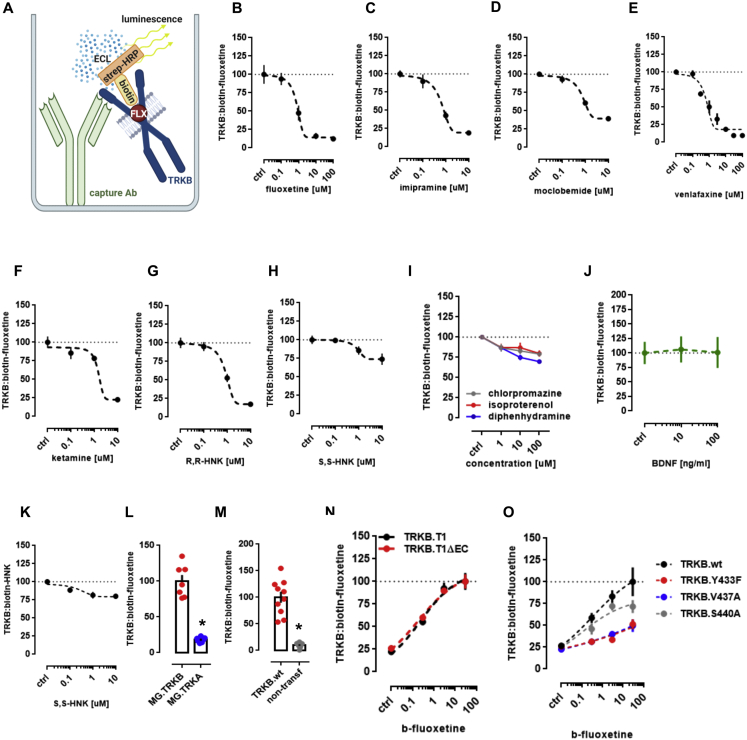
Antidepressants bind to TRKB transmembrane domain, related to Figure 2 Lysate from HEK293T cells expressing TRKB were submitted to ligand binding assays. (A) Schematic representation of the biotinylated fluoxetine interaction with immobilized TRKB. (B-J) Biotinylated fluoxetine (1 μM) interaction with TRKB is reduced by non-biotinylated (B) fluoxetine (n = 6/group), (C) imipramine (n = 8/group), (D) moclobemide (n = 10/group), (E) venlafaxine (n = 6/group), (F) ketamine (n = 8/group), (G) R,R-HNK (n = 8/group), but not reduced by (H) S,S-HNK (n = 8/group), (I) chlorpromazine (n = 8/group), isoproterenol (n = 8/group) or diphenhydramine (n = 8/group), or (J) BDNF (n = 6/group). (K) Biotinylated R,R-HNK (1 μM) interaction with TRKB is not reduced by S,S-HNK (n = 12/group). (L,M) Biotinylated fluoxetine interaction with (L) TRKA (n = 7/group) from MG87 cells, or (M) lysates from non-transfected HEK cells (n = 10/group) are negligible compared to TRKB. The interaction of biotinylated fluoxetine is not altered in (N) TRKB lacking most of the intra and extracellular domains (TRKB.T1ΔEC, n = 12/group), but it is reduced by (O) V437A and Y433F mutations, and partially attenuated by S440A (n = 6/group).

We then tested if ADs directly bind to TRKB. First, we found that biotinylated FLX binds to immunoprecipitated TRKB with a low μM affinity (K_d_ = 2.42 μM) (Figure 2C), but not to TRKA or lysates from non-transfected cells (Figures S4L and S4M). Although the affinity of FLX to serotonin transporter (5HTT) is much higher than that to TRKB, micromolar affinity corresponds well to the concentrations of ADs reached in the human brain during chronic treatment (Bolo et al., 2000; Henry et al., 2000; Johnson et al., 2007; Karson et al., 1993). Binding of biotinylated FLX (1 μM) to TRKB was displaced by unlabeled FLX (K_i_ = 1.69 μM) (Figure S4B), indicating specific binding. A deletion construct without most of the extracellular and intracellular domains of TRKB except for the TMD and short juxtamembrane sequences (TRKB.T1ΔEC) (Haapasalo et al., 1999) also demonstrated robust binding (Figure S4N), whereas FLX failed to bind to a chimeric TRKB with the TMD of TRKA (Figure 2D), which focuses the binding activity to the TRKB TMD.

**Figure S5:**
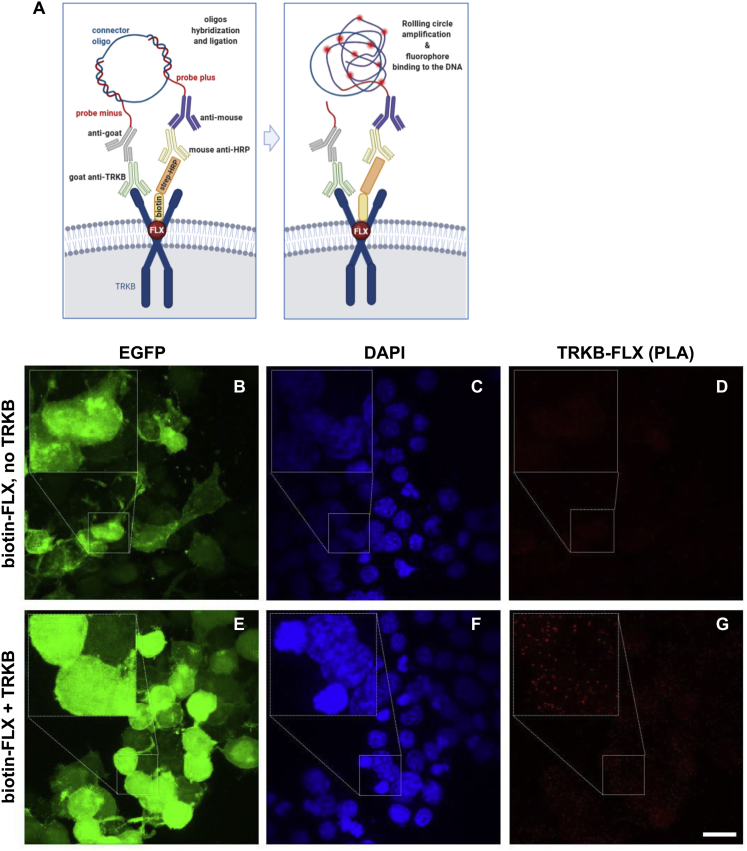
Antidepressants bind to TRKB in the intact cells, related to Figure 2 (A) Schematic representation of the *in situ* proximity ligation assay (PLA) between TRKB and biotinylated fluoxetine. (B-G) HEK cells were transfected to express TRKB and farnesylated GFP and were exposed to biotinylated fluoxetine (10uM/15min). The cells were fixed in PFA and the PLA reaction was conducted in permeabilized cells. (B-D) No signal from TRKB-FLX interaction was observed when cells were not transfected to express TRKB. (E-G) positive signal of TRKB-FLX (PLA). Scale bar: 20 μm. Zoom in square: 2.5x.

Binding of FLX to TRKB was also observed in intact cells using *in situ* proximity ligation assay (PLA). A robust PLA signal was observed when N2A cells transfected to express TRKB were exposed to biotinylated FLX (Figures 2J and S5E–S5G), confirming a close proximity of bound FLX to TRKB. No signal was observed with FLX in control cells lacking TRKB (Figures 2K and S5B–S5D).

To verify the direct interaction between FLX and TRKB, we used MST assay (Jerabek-Willemsen et al., 2014) that detects ligand-receptor binding directly in cell lysates (Welsch et al., 2017). This assay confirmed that unlabeled FLX directly binds to GFP-tagged TRKB in lysates of transfected HEK293T cells (Figure 2L).

We further found that tritiated imipramine binds to TRKB at micromolar affinity (K_d_ = 1.43 μM) (Figure 2F), similar to that seen with FLX. Binding of biotinylated FLX (1 μM) to TRKB was displaced by imipramine, venlafaxine, moclobemide, KET, esketamine, and R,R-HNK with K_i_ of 1.03, 2.08, 1.51, 12.30, 2.86, and 2.23 μM, respectively (Figures 2G and S4C–S4G). By contrast, control compounds isoproterenol, chlorpromazine, diphenhydramine, and 2S,6S-HNK that are structurally and physico-chemically similar to ADs, produced weak, if any, displacement of biotinylated FLX (Figures S4H and S4I). BDNF failed to displace FLX from TRKB (Figure S4J), which is consistent with different interaction sites.

The finding that KET and R,R-HNK compete with FLX indicates that not only the typical but also the novel rapid-acting ADs bind to TRKB. Remarkably, R,R-HNK clearly binds to TRKB (K_d_ = 1.82 μM) (Figure 2E), and S,S-HNK failed to displace bound R,R,-HNK, indicating that AD binding to TRKB is stereoselective (Figure S4K). R,R-HNK produces AD-like effects in rodents at concentrations that do not inhibit NMDA receptors, the proposed primary interaction site for rapid-acting ADs (Zanos et al., 2016, 2018), but no alternative binding site for R,R-HNK that could explain its AD-like effects has been identified. Our finding suggests that TRKB might be this elusive direct target for R,R-HNK. CHOL did not compete with FLX or R,R-HNK, but increased the interaction of these compounds with TRKB, suggesting the presence of two distinct and cooperative recognition mechanisms for CHOL and ADs (Figures 2H and 2I).

These acute effects of ADs are not mediated by functional inhibition of acid sphingomyelinase (FIASMA) or sphingolipid metabolism (Kornhuber et al., 2010), because FIASMA compounds chlorpromazine, pimozide, and flupenthixol failed to rescue BDNF-induced activation of TRKB under high concentrations of CHOL, as FLX does (Figures 2B and S2H). Further, chlorpromazine failed to displace FLX binding to TRKB (Figure S4I), whereas non-FIASMA ADs venlafaxine, KET, and R,R-HNK readily displaced FLX (Figures S4E–S4G). Together, these results suggest that all of the investigated ADs directly bind to TRKB at clinically meaningful concentrations (Bolo et al., 2000; Henry et al., 2000; Karson et al., 1993).

### Modeling fluoxetine binding to TRKB

**Figure 3:**
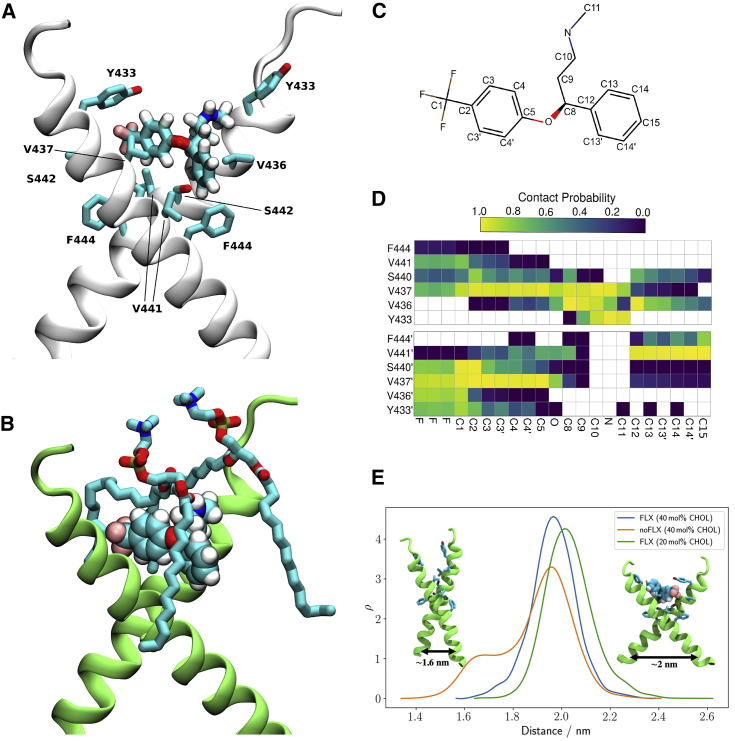
Model of fluoxetine interaction with TRKB transmembrane domain The fluoxetine binding pocket at the dimeric interface of the TRKB transmembrane helices. (A) A representative snapshot showing fluoxetine in the crevice between the TRKB monomers. Fluoxetine is shown in licorice and the protein in cartoon representations. The side chains that interact with the drug are labeled and shown in licorice. (B) Fluoxetine binding involves lipid molecules, which provide a closed cavity for the drug. The protein is shown in green cartoon, the drug in van der Waals, and the lipids in licorice representations. (C) The chemical structure of fluoxetine. The atom names are labeled and the chemically equivalent atoms are indicated with an apostrophe. (D) The contact probability between drug heavy atoms and the interacting protein residues. The upper and lower panels correspond to the two different transmembrane helices (residues of the second helix are tagged with an apostrophe). Contact probabilities are calculated using a minimum distance cutoff of 5 Å (system 10). (E) The distributions of the distance between the center of mass L451–L453 Cα atoms of each monomer are shown for membranes with 20 mol % cholesterol (green; system 9), 40 mol % cholesterol with (blue; system 10) and without bound FLX (orange; system 7). See also Figures S3 and S6 and Table S1.

Docking followed by an extensive set of 120 1-μs-long MD simulations suggested a binding site and mode for FLX in the crevice facing the extracellular side of the crossed TRKB TMD dimer (Figure 3A). This binding site and mode engages both TMDs in the dimer and also recruits phospholipids, which can further stabilize the binding (Figure 3B). The simulations also revealed several protein residues important for binding, including Y433, V437, and S440 (Figures 3A, 3C, and 3D). Mutagenesis experimentally verified this binding site: FLX binding to TRKB.Y433F and TRKB.V437A was essentially lost, and binding to TRKB.S440A was significantly reduced (Figures 2C and S4O). Furthermore, binding of FLX to a chimeric TRKB carrying TMD from TRKA (TRKB/TRKA.TM) was very low, and the affinity of imipramine and R,R-HNK to TRKB.Y433F was also much lower than to the wild-type TRKB (Figures 2D–2F).

As shown above, the stable configuration of TRKB TM dimers at 20 mol % of CHOL is destabilized by increased membrane thickness at 40 mol % of CHOL (Figure 1G,H). Remarkably, at 40 mol %, FLX binding maintained the active configuration of TRKB dimers close to that observed in 20 mol % (Figure 3E), which is consistent with our biochemical observation that FLX preferentially acts under high CHOL conditions (Figure 2B). Following drug expulsion, the dimers transitioned to the more parallel conformation seen in Figure 1I. Protein-free simulations with varying CHOL concentrations showed that interaction of ADs with membrane lipids alone does not explain the observed drug binding, because neither FLX, R,R-HNK, nor esketamine altered the order parameters of the membrane lipids (systems 21–50 in Table S1). The Y433F mutation decreased the residence-time of FLX by at least 4-fold, to 161 ns when compared to the >696 ns for the wild-type protein, consistent with a low binding affinity of FLX to TRKB.Y433F (Figure 2C). Accordingly, Y433 is directly involved in FLX binding and indirectly involved in CHOL sensing via membrane thickness. The other mutations (V437A, S440A; Table S1, systems 12–14) also substantially decreased the FLX residence-time and binding affinity (Figure S4O). Together, these data suggest that FLX, by binding to the dimeric TRKB interface, acts like a wedge and stabilizes the cross-shaped active conformation at high CHOL concentration typically present in synaptic membranes (Figures 3A and 3E).

### Antidepressants promote membrane trafficking of TRKB

**Figure 4:**
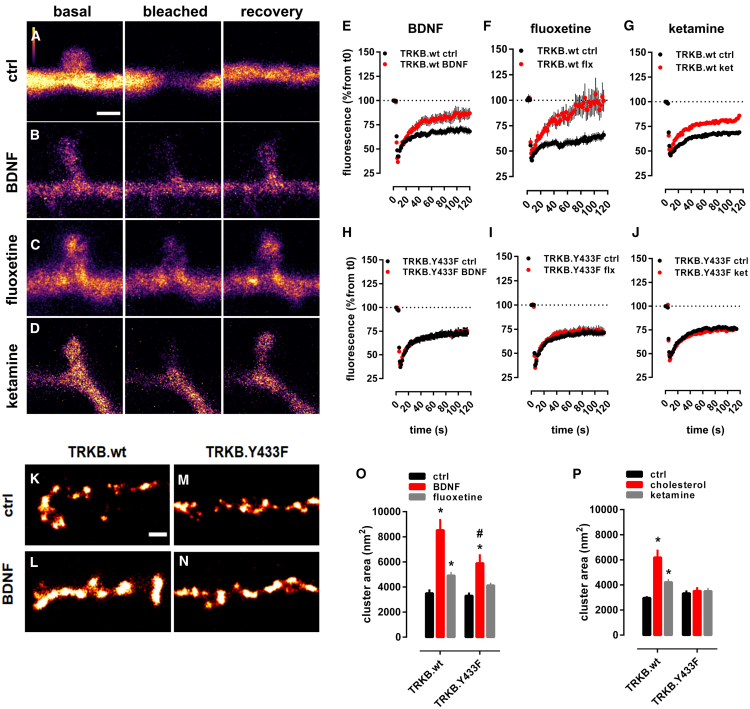
Antidepressants promote membrane trafficking of TRKB (A–D) Representative images of the spine and shaft fluorescence in (A) control, (B) BDNF-, (C) fluoxetine-, or (D) ketamine-treated rat hippocampal neurons (E18; DIV14) transfected with GFP-TRKB before (basal), immediately (bleached), and 2 min (recovery) after photobleaching (for analysis of neurite shaft recovery, see Figure S4A). Scale bar, 1,000 nm. (E–J) Recovery of GFP-TRKB in dendritic spines is increased by (E and H) BDNF (20 ng/mL/15 min, TRKB.wt n = 17–27; interaction: F[62,2,604] = 5.435, p = 0.0001; TRKB.Y433F n = 27–39; interaction: F[52,3,328] = 0.4595, p = 0.99), (F and I) fluoxetine (1 μM/15 min, TRKB.wt n = 9–22; interaction: F[177,3,068] = 2.220, p = 0.0001; TRKB.Y433F n = 28–42; interaction: F[59,4,012] = 0.5555, p = 0.99), and (G and J) ketamine (10 μM/15 min, TRKB.wt n = 15–18; interaction: F[59,1,829] = 3.361, p < 0.0001; TRKB.Y433F n = 20–22; interaction: F[59,2,360] = 0.3995, p > 0.9999), but this is prevented in GFP-TRKB.Y433F expressing neurons; data expressed as mean ± SEM of percentage from t = 0. (K–N) Representative images of the BDNF-induced clusters of GFP-TRKB on the surface of MG87.TRKB cells. Scale bar, 250 nm. (O and P) BDNF (10 ng/mL/15 min) and fluoxetine (10 μM/15 min, TRKB.wt n = 365–593; TRKB.Y433F n = 232–547; interaction: F[2,2,717] = 4.305, p = 0.0136) (O) and cholesterol (20 μM/15 min) and ketamine (10 μM/15 min, TRKB.wt n = 282–7,413; TRKB.Y433F n = 258–765; interaction: F[2,2,731] = 11.15, p < 0.0001) (P) enhance the formation of clusters of GFP-TRKB on the surface of MG87.TRKB cells but not in the GFP-TRKB.Y433F-expressing cells. ^∗^p < 0.05 from respective control (vehicle-treated) groups; #p < 0.05 from BDNF- or fluoxetine-treated wild-type group (Fisher’s LSD), clusters from 10 cells/group, and 10 regions of interest (ROI) per image, mean ± SEM of cluster area (nm^2^). See also Figure S6A.

**Figure S6:**
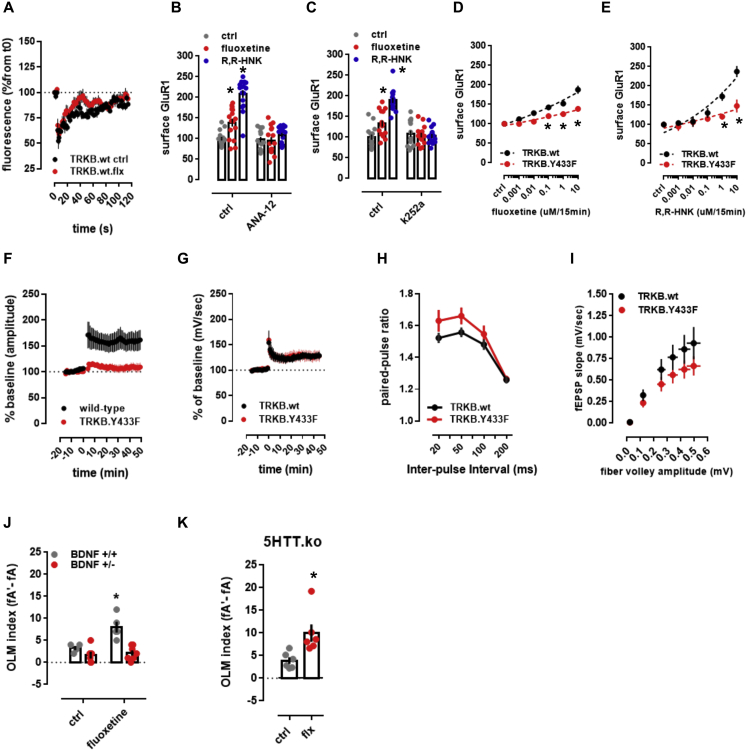
Antidepressants and cholesterol promote membrane trafficking and TRKB-mediated plasticity, related to Figures 4 and 5 (A) The fluorescence recovered after bleaching of GFP-TRKB in the neurite shaft of hippocampal neurons [n = 4-6; interaction: F(59,480) = 0.7580, p = 0.9061]. (B-E) Fluoxetine- and R,R-HNK-induced increase in the surface levels of GluR1 subunit of AMPA receptors are prevented by (B) ANA-12 [F(2,89) = 22.13, p < 0.0001, n = 15-16], (C) k252a [F(2,89) = 27.83, p < 0.0001, n = 15-16] in rat cortical cells, and by (D,E) the Y433F mutation of TRKB [fluoxetine: F(5,132) = 3.941, p = 0.0023, n = 12/group; R,R-HNK: F(5,132) = 5.022, p = 0.0003, n = 12/group] in mouse cortical cells. Data expressed as mean ± SEM of percentage from ctrl group. ^∗^p < 0.05 from ctrl/TRKB.wt at the same dose. (F-I) Electrophysiological parameters of TRKB.Y433F mice. (F) TRKB.Y433F mutant mice display reduced theta-burst stimulus-induced changes in LTP [n = 6/group; interaction: F(61,610) = 5.466; p < 0.0001] but no changes in the (G) tetanic-stimulus-induced LTP [n = 5/group; interaction: F(60,480) = 0.1333, p > 0.9999], although a significant genotype effect was observed in (H) paired-pulse facilitation [n = 9/group; genotype: F(1,64) = 5.664, p = 0.0203; interaction: F(3,64) = 0.6356, p = 0.5948] and (I) input-output ratio [n = 9/group; genotype: F(1,96) = 6.388, p = 0.0131; interaction: F(5,96) = 0.3945, p = 0.8515] no interaction was identified. Data expressed as mean ± SEM of percentage from t0, baseline, or ctrl group. (J-K) Fluoxetine-induced (15mg/kg/7days in drinking water) increased performance in OLM was prevented in mice (J) heterozygous to BDNF [n = 4-7; interaction: F(1,18) = 6.878, p = 0.0173], but not in (K) animals lacking the serotonin transporter [n = 6/group; t(10) = 2.962, p = 0.0142]. ^∗^p < 0.05 from ctrl.

We used fluorescence recovery after photobleaching (FRAP) assay in primary hippocampal neurons (DIV14) to evaluate the mobility of TRKB in neuronal spines. In neurons transfected to express GFP-tagged TRKB, the fluorescence was rapidly recovered in dendritic shafts, but not in spines after bleaching (Figures 4A and S6A). Pretreatment with BDNF (20 ng/mL/15 min) brought about a rapid recovery of GFP-TRKB fluorescence in spines after bleaching, indicating TRKB trafficking to spines (Figures 4B and 4E). Similarly, pretreatment of neurons with FLX (1 μM/15 min) or KET (10 μM/15 min) also promoted recovery of GFP-TRKB fluorescence in dendritic spines (Figures 4C, 4D, 4F, 4G, and S6A) without any additional effect on dendritic shafts. Neither BDNF, FLX, nor KET increased the fluorescence of GFP-TRKB.Y433F mutant receptors in dendritic spines after bleaching (Figures 4H–4J), although the localization of GFP-TRKB.Y433F before bleaching was identical to the wild-type GFP-TRKB. These data demonstrate that BDNF, FLX, and KET promote TRKB trafficking in dendritic spines, and this effect is disrupted in TRKB.Y433F mutants.

Super-resolution microscopy (dSTORM/TIRF) revealed that BDNF, FLX, KET, and CHOL all increased the size of clusters formed by wild-type GFP-TRKB, but not clusters of GFP-TRKB.Y433F mutants at the plasma membrane of fibroblast cell line, indicating that the increased trafficking may lead to increased cell surface expression and clustering of TRKB (Figures 4K–4P). The basal cell surface expression of GFP-TRKB.Y433F was again similar to that of the wild-type TRKB (Figures 4K, 4M, and 4O).

ADs are known to increase the cell surface expression of AMPA glutamate receptors and the blockade of AMPA receptors prevents the behavioral effects of KET and R,R-HNK (Maeng et al., 2008; Zanos et al., 2016). We confirmed that FLX and R,R-HNK increased cell surface localization of GluR1 subunits of AMPA receptors, and this effect was prevented by the TRKB inhibitors ANA12 and k252a (Figures S6B and S6C) and in neurons from TRKB.Y433F mutant mice (Figures S6D and S6E). These data suggest that the effect of ADs on synaptic AMPA receptor surface exposure is a downstream effect of TRKB activation by these drugs.

### Binding to TRKB mediates antidepressant-induced plasticity

BDNF is a critical mediator of synaptic plasticity and is required for long-term potentiation (LTP) in slices as well as *in vivo*, and these effects are mediated by TRKB (Ernfors and Bramham, 2003; Minichiello, 2009; Panja and Bramham, 2014). Theta-burst stimulation reliably induced an LTP in the CA3-CA1 synapses in slices derived from wild-type mice. Remarkably, similar stimulation of slices derived from heterozygous mice carrying a TRKB.Y433F mutation (TRKB.Y433F mice) failed to induce any significant potentiation (Figure S6F). However, tetanic stimulation induced LTP in both wild-type and TRKB.Y433F slices (Figure S6G), consistent with the central role of BDNF in theta-burst-mediated LTP (Kang et al., 1997; Minichiello et al., 2002; Patterson et al., 2001).

**Figure 5:**
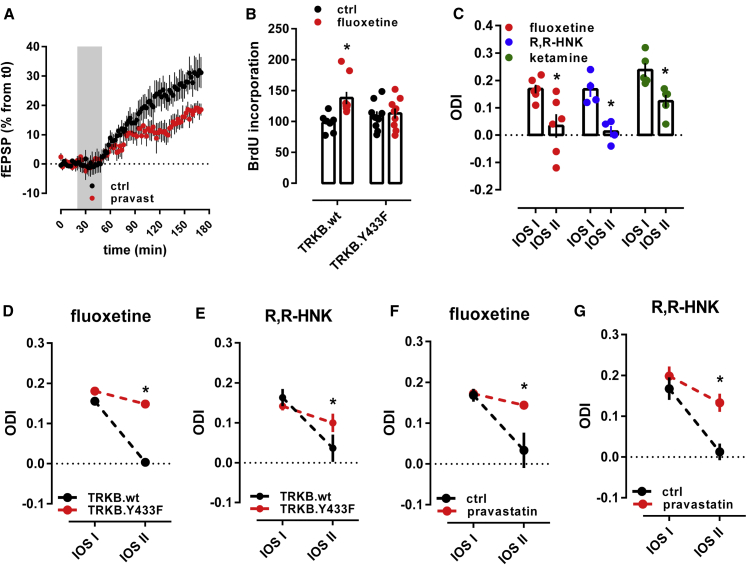
Binding to TRKB mediates the plasticity-related effects of antidepressants (A) Treatment with pravastatin (10 mg/kg/day in the drinking water for 14 days) attenuated the BDNF-induced LTP in the hippocampus of anesthetized rats (F[85,1,290] = 1.484, p = 0.0036, n = 8–9). (B) Fluoxetine promotes hippocampal neurogenesis in wild-type, but not in TRKB.Y433F mice (n = 7–9; interaction: F[1,30] = 4.691, p = 0.0384). Mice received bromodeoxyuridine (BrdU) injections at day 1, the BrdU incorporation was measured after 3 weeks of fluoxetine treatment (15 mg/kg/day for 21 days in the drinking water, orally [p.o.]). (C) Fluoxetine (10 mg/kg/day for 28 days, p.o.; n = 6), R,R-HNK (10 mg/kg i.p. injection every second day for 8 days, n = 4), and ketamine (10 mg/kg i.p. injection every second day for 8 days, n = 5) permitted a shift in ocular dominance in adult mice during 7 days of monocular deprivation (paired t test: fluoxetine: t[5] = 2.985, p = 0.0306; R,R-HNK: t[3] = 6.875, p = 0.0063; ketamine: t[4] = 6.517, p = 0.0029). *p < 0.05 between intrinsic signal imaging (IOS) sessions. (D and E) Fluoxetine (D) and R,R-HNK (E) fail to permit a shift in ocular dominance in TRKB.Y433F mice (fluoxetine: F[1,19] = 256.9, p < 0.0001, n = 9–12; R,R-HNK: F[1,20] = 12.47, p = 0.0021, n = 6/group). (F) Treatment with fluoxetine induced a shift in ocular dominance in response to 7 days of monocular deprivation, but this effect is prevented by pravastatin (interaction: F[1,10] = 5.221, p = 0.0454, n = 5–6). (G) R,R-HNK induced a shift in ocular dominance in response to 7 days of monocular deprivation, but this effect is prevented by pravastatin (treatment: F[1,9] = 9.044; p = 0.0148, n = 4–7). ^∗^p < 0.05 from the control group in the same session, Fisher’s LSD. Data expressed as mean ± SEM. The black groups in plots (F) and (G) are also depicted in (C). See also Table S2 and Figure S6.

Infusion of BDNF into the dentate gyrus of anesthetized rats significantly increased synaptic strength, as previously reported (Messaoudi et al., 2002; Panja and Bramham, 2014). However, this effect of BDNF was partially prevented when rats were co-treated with pravastatin (10 mg/kg/day/14 days) (Figure 5A), suggesting that neuronal CHOL is required for the effects of BDNF on LTP.

ADs increase the proliferation and survival of newly born dentate granule neurons (Malberg et al., 2000; Sairanen et al., 2005; Santarelli et al., 2003). We confirmed that FLX (15 mg/kg/day, leading to FLX brain concentration of 31.9 ± 5.9 μM) (Table S2) significantly increased survival of newborn hippocampal neurons in wild-type mice, however, no increase was observed in the dentate gyrus of TRKB.Y433F mice (Figure 5B).

Chronic FLX treatment reactivates critical period-like plasticity in the visual cortex of adult mice, allowing an ocular dominance (OD) shift in response to monocular deprivation, which normally only happens during a developmental critical period (Maya Vetencourt et al., 2008; Steinzeig et al., 2017). In mice treated with FLX for 4 weeks (10 mg/kg/day), a 7-day monocular deprivation during the last treatment week induced a dramatic shift in OD in favor of the open eye (Figure 5C). We now show that both KET and R,R-HNK (both 10 mg/kg, intraperitoneal [i.p.]) also induced a significant shift in OD, but a much shorter treatment was needed than that for FLX (Figure 5C), consistent with their fast action. The response to R,R-HNK was comparable to that produced by FLX, however, the magnitude of response to KET was lower than that to FLX and R,R-HNK. Remarkably, the effect of FLX and R,R-HNK on the shift in ocular dominance was lost in TRKB.Y433F mice (Figures 5D and 5E) and in wild-type mice co-treated with pravastatin (Figures 5F and 5G), indicating that the plasticity-inducing effects of ADs may be mediated by their direct binding to TRKB.

### Binding to TRKB mediates the behavioral effects of antidepressants

**Figure 6:**
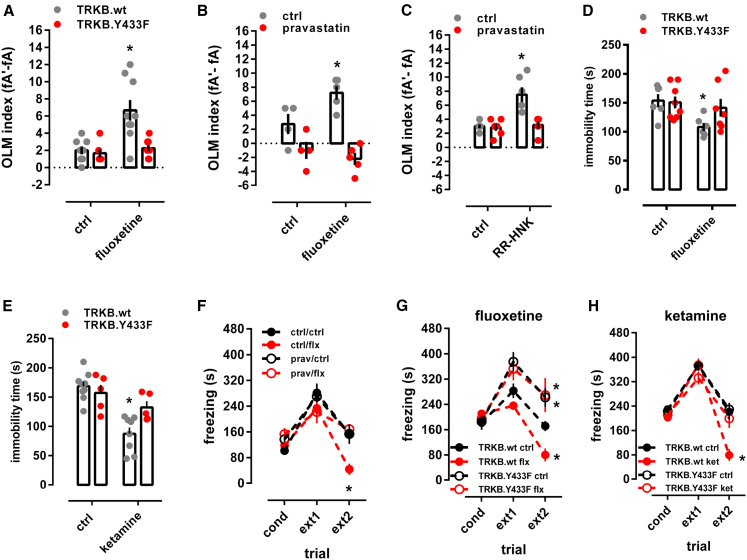
Binding to TRKB mediates the behavioral effects of antidepressants (A) Fluoxetine improves object location memory (OLM) in wild-type mice, but this effect was absent in the TRKB.Y433F mice (interaction: F[1,18] = 6.878, p = 0.017; n = 8–9). (B) Fluoxetine improved object location memory in wild-type mice, but this effect was prevented by pravastatin (interaction: F[1,14] = 6.504, p = 0.023, n = 4-5). (C) R,R-HNK improved object location memory in wild-type mice, but this effect was prevented by pravastatin (interaction: F[1,20] = 10.59, p = 0.0040, n = 6/group). (D and E) Fluoxetine (D) (treatment: F[1,23] = 5.433, p = 0.0289, n = 6–8) and ketamine (E) (treatment: F[1,23] = 24.26, p < 0.0001, n = 5–9) reduce immobility in the forced swimming test in TRKB.wt mice, but are ineffective in TRKB.Y433F mutants. (F) Fluoxetine facilitated the extinction of contextual conditioned fear, and this response is blocked by pravastatin (interaction: F[6,40] = 5.099, p = 0.0006, n = 6/group). (G and H) Fluoxetine (G) and ketamine (H) facilitate the extinction of contextual conditioned fear in the 8-min session, and this response is blocked in mice carrying the TRKB.Y433F mutation (fluoxetine: F[6,34] = 3.241, p = 0.0126; n = 5–6; ketamine: F[6,40] = 4.896, p = 0.0008; n = 5–7). ^∗^p < 0.05 from the control group in the same session, Fisher’s LSD. Data expressed as mean ± SEM. See also Figure S6.

We next investigated whether AD interaction with TRKB influences neuronal plasticity-dependent learning and behavior. FLX (15 mg/kg/day) for 7 days facilitated long-term memory in object location memory (OLM) test in TRKB.wt mice, but not in TRKB.Y433F mice, although the behavior of vehicle-treated TRKB.Y433F mice was similar to their vehicle-treated wild-type littermates (Figure 6A). A similar lack of response to FLX was observed in BDNF haploinsufficient mice (Figure S6J) and in animals co-treated with pravastatin (Figures 6B and 6C). Remarkably, serotonin transporter knockout (5HTT.ko) mice lacking the primary site of action of SSRIs responded to FLX treatment normally in the OLM test (Figure S6K), indicating that the effects of FLX in this test are not mediated by inhibition of serotonin transport. This is consistent with the findings that the biochemical, behavioral, and electrophysiological effects of SSRIs are preserved in 5HTT.ko mice (Normann et al., 2018; Rantamäki et al., 2011). However, a recent study found that behavioral effects of FLX are lost in mice with a point mutation in 5HTT that impairs the response to AD drugs (Nackenoff et al., 2016).

BDNF-TRKB signaling is known to be sufficient and necessary for the effects of ADs in the forced swimming test (FST) (Koponen et al., 2005; Monteggia et al., 2004; Saarelainen et al., 2003). FLX (15 mg/kg, for 21 days) and KET (10 mg/kg i.p., 2 h before) significantly reduced immobility in the FST in wild-type mice, but both drugs were ineffective in TRKB.Y433F mice (Figures 6D and 6E).

FLX promotes extinction of conditioned fear in a BDNF-dependent manner (Andero and Ressler, 2012; Deschaux et al., 2011; Karpova et al., 2011). The freezing response was indistinguishable between the genotypes immediately after conditioning (Figures 6G and 6H). FLX (15 mg/kg for 2 weeks, starting immediately after conditioning) and KET (10 mg/kg i.p., immediately after conditioning and 2 h before each extinction trial) promoted extinction of conditioned fear in wild-type mice (Figures 6G and 6H), but no increase in fear extinction was seen in FLX- or KET-treated TRKB.Y433F mice (Figures 6G and 6H) or in pravastatin-FLX co-treated mice (Figure 6F).

Together, these data demonstrate that the behavioral effects produced by different ADs were lost in TRKB.Y433F mutant mice, consistently with AD binding to TRKB. Moreover, we observed that many of these behavioral effects were also lost when ADs were co-administered with pravastatin (Figures 6B, 6C, and 6F), supporting the notion that CHOL sensing is necessary for the behavioral effects of BDNF on TRKB signaling.

## Discussion

### Antidepressants bind to TRKB

BDNF signaling is crucial for the action of essentially all AD drugs (Autry and Monteggia, 2012; Castrén and Antila, 2017; Duman and Monteggia, 2006), but this effect has been assumed to be indirectly mediated by other proteins such as 5HTT or NMDA receptors. We now show that ADs bind to the TMD of TRKB dimers with a therapeutically relevant affinity (Bolo et al., 2000; Karson et al., 1993), stabilizing a conformation of the TRKB TM dimers favorable for signaling, thereby promoting TRKB translocation to and retention at the plasma membrane, where it is accessible to BDNF. Specific binding was observed not only for FLX and imipramine, representing typical SSRI and TCAs, respectively, but also for the rapid-acting KET metabolite RR-HNK. Binding of labeled FLX was displaced by FLX itself and by imipramine, moclobemide, RR-HNK, KET, and esketamine, which suggests that these drugs bind to at least partially overlapping sites. These data suggest that direct interaction with the TMDs of TRKB dimer may function as a binding site for several different, if not all, ADs.

MD simulations identified a binding site for FLX at the outer opening of the crossed dimer of TRKB TMDs. Several mutations predicted by and tested in MD were used in experimental mutagenesis, which confirmed the binding site. Our data suggest that in thick CHOL-rich membranes, typically found in synapses and lipid rafts, dimers of TRKB TMDs assume a near-parallel, presumably unstable position, which leads to the exclusion of TRKB from synaptic membranes and limits synaptic TRKB signaling (Pereira and Chao, 2007; Suzuki et al., 2004). Binding of FLX to a site formed by the crossed TMDs acts as a wedge, maintaining a more stable structure in synaptic membranes, thereby allosterically facilitating synaptic BDNF signaling. Simulations predict that membrane lipids also participate in FLX binding to TRKB. Because TRKB exists as a multi-protein complex that also includes transmembrane proteins (Fred et al., 2019; Lesnikova et al., 2020), it is possible that other proteins and lipids participate in AD binding to TRKB in cell-type- and subcellular compartment-dependent manner. Further characterization of this binding site may yield important information for discovery of new ADs with increased potency for plasticity-related behavioral effects.

It has been known for decades that the clinical response to typical ADs is only reached after several days or weeks of treatment, but the reason for this delay has remained a mystery. One explanation has been that the process of neuronal plasticity induced by ADs may take time to develop. However, the discovery of the rapid action of KET, which is also dependent on plasticity, has undermined this explanation. FLX and imipramine bind to 5HTT with a much higher affinity than to TRKB, while the affinity of KET to TRKB is comparable to its affinity to NMDA receptors (Zanos et al., 2018). Remarkably, micromolar concentrations of typical ADs are reached and required in the brain during chronic treatment, as shown in humans for FLX (Bolo et al., 2000; Henry et al., 2000; Johnson et al., 2007; Karson et al., 1993), fluvoxamine (Bolo et al., 2000), and paroxetine (Henry et al., 2000) and here for FLX in mice. Importantly, typical ADs gradually accumulate in the brain, reaching a plateau after several weeks of treatment (Karson et al., 1993; Kornhuber et al., 1995), suggesting that the clinical response is only achieved when the drug reaches a brain concentration high enough to interact with a low-affinity binding target, such as TRKB. Sufficient concentrations may not be reached in fast metabolizers or patients with limited compliance, which may contribute to the failure to respond. KET, on the other hand, readily penetrates to the brain to achieve sufficient synaptic concentrations quickly. Therefore, the gradual increase in brain concentration to a level needed for TRKB binding might be at least one explanation for why typical ADs take so long to act, while the rapid brain penetration of KET enables fast action. Nevertheless, it is unlikely that effects on TRKB mediate all the effects of ADs and that inhibition of 5HTT and NMDA receptors also play a role (Harmer et al., 2017).

A previous study reported binding of amitriptyline, but not many other ADs, to the extracellular domains of TRKB and TRKA (Jang et al., 2009). AD binding detected here is clearly distinct from that amitriptyline binding, as it includes many different ADs, it is specific to TRKB, and TRKB construct including the TMD but lacking the reported amitriptyline binding site in the first leucine-rich repeat readily binds FLX.

Our findings imply that high doses of statins might interfere with the AD response. A recent study indicates more depression and more AD use among statin users (Köhler-Forsberg et al., 2019), but meta-analyses have found, if anything, less depression among statin users (Yatham et al., 2019). This discrepancy to our rodent findings is likely related to the high statin dose used in our studies. Interestingly, serum CHOL levels have been found to be low in suicidal patients (Kim and Myint, 2004).

Due to the effects of TRKB on neuronal survival and plasticity, small-molecule agonists of TRKB have been actively searched (Longo and Massa, 2013; Saragovi et al., 2019). Our data show that ADs bind to TRKB and allosterically potentiate BDNF signaling, thereby maintaining use-dependency, which limits the action of TRKB selectively to active synapses that release BDNF, avoiding undesirable stabilization of inactive synapses in a way full TRKB agonists may do. This action of ADs as “smart drugs” is consistent with the wide utility of these drugs in many neurological and psychiatric disorders beyond depression (Schneider et al., 2019).

### TRKB-cholesterol interaction

Astrocyte-derived CHOL has been recognized as an important regulator of neuronal maturation and plasticity (Martin et al., 2014; Mauch et al., 2001; Pfrieger and Ungerer, 2011), but the mechanisms through which CHOL acts to produce these effects have remained unclear. Here, we have demonstrated that TRKB TMD possesses a CARC domain (Fantini and Barrantes, 2013), and CHOL potentiates the effects of BDNF on TRKB signaling.

CHOL regulates BDNF signaling (Pereira and Chao, 2007; Suzuki et al., 2004; Zonta and Minichiello, 2013), and BDNF, in turn, promotes neuronal CHOL synthesis (Suzuki et al., 2007). Synaptic membranes are enriched in CHOL and resemble CHOL-rich lipid rafts (Ikonen, 2008). TRKB normally resides outside rafts but can transiently translocate to rafts upon BDNF stimulation (Pereira and Chao, 2007; Suzuki et al., 2004), as also observed here. This translocation may be related to our observation of TRKB trafficking to dendritic spines and clustering on the plasma membrane, both of which were stimulated by BDNF and ADs. TRKB residence in lipid rafts is short-lived (Pereira and Chao, 2007; Suzuki et al., 2004), which may be explained by our simulation data suggesting instability of the crossed TRKB TMD structure in thick CHOL-rich membranes. Translocation of TRKB to rafts is dependent on FYN kinase (Pereira and Chao, 2007; Suzuki et al., 2004), and we observed that BDNF increases interaction of FYN with wild-type TRKB, but not with the TRKB.Y433F mutant cells. These data suggest a scenario where the interaction between TRKB and BDNF or ADs promotes its retention in CHOL-rich synaptic membranes.

Our simulation data predict that TMDs of TRKB interact at ^439^AXXXG^443^ dimerization motif, and suggest that, analogous to the EGF receptor (Arkhipov et al., 2013; Endres et al., 2013; Sinclair et al., 2018), the angle between the dimerized and crossed TRKB TMDs, regulated by the CHOL-regulated membrane thickness, plays an important role in TRKB signaling. Obviously, the configuration of the TMD is not the only determinant of TRKB signaling capacity, nevertheless, our findings are a major step forward in understanding the interaction of TRKB with cellular membranes.

TRKB appears to be the only CHOL-sensing member from the TRK family of neurotrophin receptors. Although TRK family members show high homology, the TMD of TRKB differs from that of TRKA and TRKC, which, in contrast to TRKB, act as dependence receptors inducing cell death in the absence of a ligand (Nikoletopoulou et al., 2010); this property is apparently dependent on the transmembrane domain (Dekkers et al., 2013). Our data suggest that TRKB has evolved to become a CHOL sensor, which may be important for its function as mediator of activity-dependent plasticity.

### Conclusions

The present findings demonstrate that ADs bind to TRKB and allosterically increase BDNF signaling, thereby directly linking the effects of ADs to neuronal plasticity. AD-induced plasticity is utilized by network-specific neuronal activity to guide re-wiring of plastic networks, allowing beneficial re-adaptation of networks abnormally wired during development or by stress (Castrén, 2013). Our data suggest a framework that unites the effects of all ADs with therapy-mediated guidance to achieve the clinical AD response.

## STAR★methods

### KEY RESOURCES TABLE

**Table:** 

REAGENT or RESOURCE	SOURCE	IDENTIFIER
**Antibodies**		

anti-TRKB	R&D Systems	Cat#AF1494; RRID:AB_2155264
anti-PLC-gamma1	Cell Signaling Technologies	Cat#5690; RRID:AB_10691383
anti-TRKB-pY515	Cell Signaling Technologies	Cat#4619; RRID:AB_10235585
anti-TRKB-pY816	Cell Signaling Technologies	Cat#4168; RRID:AB_10620952
anti-phosphoY	AbD Serotec	Cat#MCA2472B
anti-actin	Santa Cruz	Cat#sc8432; RRID:AB_626630
anti-GFP	Abcam	Cat#ab290; RRID:AB_303395
anti-GFP	Abcam	Cat#ab13970; RRID:AB_300798
anti-flotilin2	Cell Signaling Technologies	Cat#3436; RRID:AB_2106572
HRP-conjugated anti-Gt IgG	Invitrogen	Cat#61-1620; RRID:AB_2533922
HRP-conjugated anti-Rb IgG	BioRad	Cat#170-5046; RRID:AB_11125757
HRP-conjugated streptavidin	Thermo-Fisher	Cat#21126
anti-FLAG	Santa Cruz	Cat#sc807-G; RRID:AB_675757
anti-HRP	Rockland Immunochemicals	Cat#200-3138-0100; RRID:AB_2611678

**Chemicals, peptides, and recombinant proteins**		

cholesterol	Sigma-Aldrich	Cat#C8667
beta-cyclodextrin	Sigma-Aldrich	Cat#C4555
pravastatin	Orion Pharma	N/A
fluoxetine	Bosche Scientific	Cat#H6995
imipramine	Sigma-Aldrich	Cat#I7379
tritiated imipramine	Perlkin-Elmer	Cat#NET576250UC
chlorpromazine	Sigma-Aldrich	Cat#C8138
isoproterenol	Tocris	Cat#1747
diphenhydramine	Tocris	Cat#3072
flupenthixol	Tocris	Cat#4057
pimozide	Tocris	Cat#0937
recombinant human BDNF	Peprotech	Cat#450-02
biotinylated rh-BDNF	Alomone Labs	Cat#B-250
NGF	Peprotech	Cat#450-01
2R,6R-HNK	Todd Gould	R,R-HNK
2S,6S-HNK	Todd Gould	S,S-HNK
ketamine	Pfizer	ketalar (50mg/ml)
S-ketamine	Esa-Korpi	esketamine
neurobasal medium	GIBCO	Cat#21103049
B27 supplement	GIBCO	Cat#17504044
DMEM	Lonza	Cat#BE12-614F
fetal calf serum	Sigma-Aldrich	Cat#F9665
Poly-L-lysine	Sigma-Aldrich	Cat#P4707

**Critical commercial assays**		

CellTiterGlo	Promega	Cat#G7571
Duolink *In Situ* Red Starter Kit Mouse/Goat	Sigma-Aldrich	Cat#DUO92103
EZ-Link NHS-PEG4 Biotinylation Kit	Thermo Scientific	Cat#21455

**Deposited data**		

All experimental data used in the present study are available in FigShare.	https://figshare.com	https://doi.org/10.6084/m9.figshare.12698012

**Experimental models: cell lines**		

cell line	HEK293T	HEK293T
cell line	NIH 3T3 stably expressing TRKB	MG87.TRKB
cell line	NIH 3T3 stably expressing TRKA	MG87.TRKA
cell line	N2A	N2A

**Experimental models: organisms/strains**		

mouse: C57BL/6NTac-Ntrk2em6006(Y433F)Tac	This article	TRKB.Y433F mice
mouse: C57BL6-RccHsd	Envigo (Harlan)	TRKB.wt mice
mouse: B6.129(Cg)-Slc6atm1Kpl/J	Taconic	SERT.ko
rat: Sprague-Dawley	Janvier	Sprague-Dawley rats

**Recombinant DNA**		

Plasmid for PCA	Henri Huttunen	GLuc1C-TRKB.wt/GLuc2C-TRKB.wt
Plasmid for PCA	Henri Huttunen	GLuc1C-TRKB.Y433F/GLuc2C-TRKB.Y433F
Plasmid for PCA	Henri Huttunen	GLuc1C-TRKB.wt/GLuc2C-FYN
Plasmid for PCA	Henri Huttunen	GLuc1C-TRKB.Y433F/GLuc2C-FYN
Plasmid to express GFP-tagged TRKB	Genscript	GFP-TRKB.wt
Plasmid to express GFP-tagged TRKB mutated at Y433	Genscript	GFP-TRKB.Y433F
Plasmid to express GFP-tagged TRKB mutated at V437	Genscript	GFP-TRKB.V437A
Plasmid to express GFP-tagged TRKB mutated at S440	Genscript	GFP-TRKB.S440A
Plasmid to express GFP-tagged TRKB where TM domain is substuted by TRKA.TM domain	Genscript	GFP-TRKB/TRKA.TM
Plasmid to express FLAG-tagged truncated TRKB (TRKB.T1)	Anna-Kaisa Haapasalo	TRKB.T1
Plasmid to express FLAG-tagged TM domain of truncated TRKB (TRKB.T1)	Anna-Kaisa Haapasalo	TRKB.T1.ΔEC

**Software and algorithms**		

GROMACS (https://linkinghub.elsevier.com/retrieve/pii/S2352711015000059)	https://github.com/ElsevierSoftwareX/SOFTX-D-15-00003	GROMACS
Graphpad Prism v6.01	https://www.graphpad.com/	Graphpad Prism
JASP	https://jasp-stats.org/	JASP

### Resource availability

#### Lead contact

Further information and requests for resources and reagents should be directed to Eero Castren (eero.castren@helsinki.fi).

#### Materials availability

Plasmids and mouse strain generated in this study are available upon a completed Material Transfer Agreement.

#### Data and code availability

Experimental data generated in the present study is available in FigShare (https://doi.org/10.6084/m9.figshare.12698012). This study did not generate new code.

### Experimental model and subject details

#### Cell culture

The cell line NIH 3T3 (mouse fibroblasts) stably transfected to express TRKB or TRKA (MG87.TRKB or MG87.TRKA, respectively) were used for *in vitro* assays (Antila et al., 2014), N2A and HEK293T cells were used to overexpress GFP-tagged TRKB for binding assays. The cells were maintained at 5% CO2, 37°C in Dulbecco’s Modified Eagle’s Medium (DMEM, containing 10% fetal calf serum, 1% penicillin/streptomycin, 1% L-glutamine, and, in the case of MG87.TRKB cells, with 400 mg/ml of G418). The cell lines used in the present study were not authenticated or genotyped for sex.

Cortex or hippocampus of E18 rat or mouse embryos were dissected and the cells plated in poly-L-lysine coated wells at a 0.5x10^6^ cells/ml density at 5% CO2, 37°C in Neurobasal medium as described in detail (Sahu et al., 2019). The cells were left undisturbed, except for medium change, for 8-22 days depending on the experimental procedure. The primary cultures were not genotyped for sex.

GFP-tagged TRKB.wt or mutants were expressed in cortical or hippocampal cells, HEK293T or in MG87.TRKB cells using Lipofectamine 2000 as transfection agent. All compounds used in pharmacological treatments were diluted in DMSO for *in vitro* experiments, except NGF and BDNF, diluted in PBS. Cholesterol was dissolved in DMSO and sonicated.

#### Animals

C57BL/6NTac-Ntrk2em6006(Y433F)Tac (TRKB.Y433F) mice were generated by introducing the Y433F point mutation into exon 12 of the *Ntrk2* gene using CRISPR/Cas9-mediated gene editing with a specific gRNA (Non-Seed_Seed-PAM: TCCTCAGG_TCTATGCCGTGG-TGG) and HDR (homology directed repair) oligonucleotide (GCAAGGTCATCAGACCTGGCTCTTTCTCTCTCCTCAGGTCTTCGCCGTGGTGGTGATTGCATCTGTGGTGGGATTCTGCCTGC). Introduction of the point mutation generates a restriction site for the BbsI restriction enzyme. The targeting strategy was based on NCBI transcript NM_001025074.2.

#### Pronucleus injections

After the administration of hormones, superovulated C57BL/6NTac females were mated with C57BL/6NTac males. One cell stage fertilized embryos were isolated from the oviducts at dpc 0.5. For microinjection, the one cell stage embryos were placed in a drop of M2 medium under mineral oil. A microinjection pipette with an approximate internal diameter of 0.5 μm (at tip) was used to inject the mixed nucleotide preparation (tracrRNA, crRNA, HDR oligo and Cas9 protein) into the pronucleus of each embryo. After recovery, 25-35 injected one cell stage embryos were transferred to one of the oviducts of 0.5 dpc, pseudopregnant NMRI females.

#### Founder analysis

The genomic DNA was extracted from biopsies and analyzed by PCR. The following templates were used as controls: H2O (ctrl1), wild-type genomic DNA (ctrl2). A two primer PCR (forward primer: GTTGAGGTTAAAGTGACCTGCTG; reverse primer: TCCTGCTGAGTTAGTCCACACTC) detects the CRISPR/Cas9-induced constitutive knock-in allele as well as potential indel modifications and the unmodified wild-type allele. To distinguish indel modifications from unmodified wild-type sequences, heteroduplex analysis (e.g., via capillary electrophoresis) has to be performed. The PCR amplicons were analyzed using a Caliper LabChip GX device. An aliquot of the PCR reaction was used to validate the presence or absence of the restriction site introduced via homology directed repair. 10U of BbsI enzyme was pipetted into the PCR reaction aliquot and incubated at the appropriate temperature. BbsI digest results in cleavage of the 493bp PCR product (HDR) in two fragments (362bp and 131bp).

The PCR product of the positive animals from the restriction analysis was subcloned for further characterization of the founder animals. 12 subclones have been prescreened for the integration of the HDR oligonucleotide by restriction with BbsI. The ratio between the total number of analyzed clones and a positive restriction result allows an estimation of the HDR mosaicism of the founder animals. Subsequently, up to 4 clones were sequenced to confirm correct integration of the HDR oligo and presence of the Y433F point mutation. Finally, the TRKB.Y433F mice were crossed with C57BL6-RccHsd (Envigo-Harlan, the Netherlands), in the University of Helsinki. All animals used from this strain were heterozygous for the Y433F mutation (TRKB.Y433F) or wild-type littermates, and genotyped after weaning.

The SERT-KO mice [line B6.129(Cg)-Slc6atm1Kpl/J] on a C57BL/6 background were purchased from Taconic (Hudson, NY, USA) and bred at the Biomedical Center, University of Freiburg. In every animal, tail biopsies were genotyped with PCR using primers from the Jackson Laboratory database to confirm homozygous knock-out. The SERT-KO mice showed bands at 210 bp, whereas the wild-type mice showed bands at 318 bp, which confirmed the gene disruption at the SERT locus. Finally, for *in vivo* electrophysiology, male adult Sprague-Dawley rats were used.

The present study used males and female mice (18-22 weeks old at the beginning of the experiments), and male Sprague-Dawley rats (15 weeks old at the beginning of the experiments). All animals used in the present study were experimentally naive before the beginning of the described experimental procedures. The animals were group housed (3-6 per cage - type ll: 552 cm2 floor area - for mice; and 3 per cage - type IV: 1500 cm2 - for rats, all from Tecniplast, Italy) and randomly allocated into the experimental groups, except when the drug administration was performed in the drinking water. All behavioral experiments were conducted in at least two separated cohorts and analyzed automatically or by experienced observers blind to the treatment/genotype conditions. The number of animals in each experiment was based on previous studies from the literature cited in the correspondent section. For *in vivo* experiments, fluoxetine and pravastatin were dissolved in the drinking water (0.08 or 0.1mg/l for fluoxetine and 0.08mg/l for pravastatin) and the volume consumed per cage was monitored every 3 days. R,R-HNK and ketamine were diluted in sterile saline for intraperitoneal injections.

All experimental protocols were approved by the local ethics committees in Finland (#ESAVI/10300/04.10.07/2016), Norway (#6159) and Germany (#G-18-88).

### Method details

#### *In silico* methods

##### Simulated systems and force fields

The protein simulated in this work included the transmembrane (TM) domains of a TRKB dimer (residues 427-459). For comparison, we also studied a TRKA dimer (residues 410-443). The lipid membranes considered were two-component bilayers composed of varying concentrations of palmitoyl-oleoyl-phosphatidylcholine (POPC) and cholesterol (CHOL). Drug binding studies in the case of TRKB were performed for fluoxetine (FLX). The effects of drugs on the physical properties of lipid membranes were studied for FLX, ketamine, and R,R-HNK. The Charmm36/m force field was used for proteins (Huang et al., 2017) and lipids (Klauda et al., 2010), the TIP3P model was used for water (Jorgensen et al., 1983), and a compatible parameter set was employed for ions (Beglov and Roux, 1994). Parameters for the drugs were generated using the CHARMM general force field (CGenFF) program (Vanommeslaeghe et al., 2012). For details, see below. Table S1 summarizes all simulations and relevant parameters.

##### Construction of the dimeric protein models

The 3D structure for a segment of mouse TRKB containing its TM domain (residues 427-459) in its monomeric form was generated using the FMAP (Folding of Membrane-Associated Peptides) server (Lomize et al., 2018). The server predicted that the residues V432-A456 form an α**-**helical TM segment.

RosettaMP docking framework (Alford et al., 2015) was used to predict the best dimer configurations to be used in atomistic molecular dynamics (MD) simulations. The orientation of the monomeric helix in a lipid membrane was determined with the OPM server (Lomize et al., 2006). To sample possible TM dimer interfaces and configurations extensively, each helix was rotated around the membrane normal at 30° intervals and simultaneously also tilted with respect to the membrane normal at 10° intervals covering a range between −30° and 30°, starting from the OPM predicted tilt angle. This procedure resulted in 2 × 6 × 12 = 144 starting configurations for docking. Rosetta MPdock tool (Alford et al., 2015) was used to generate 960 dimers for each of the 144 starting configurations, yielding a total of 138,240 TM dimer models. These models were sorted by their interface score, and the leading 10% were clustered into five groups using the Calibur clustering application (Li and Ng, 2010) based on the models’ structural similarity. The structures with the best interface score in each cluster were selected as representative models.

The initial model of the human TRKA TM (amino acids 410-443) dimer was adapted from the NMR structure (PDB:2N90). The initial position of the TRKA TM region in the lipid bilayers was determined using the PPM Server (Lomize et al., 2017).

##### Exploratory atomistic MD simulations for TRKB model selection and refinement

We initially performed an extensive set of exploratory simulations (10 repeats × 500 ns × 5 dimers × 2 membrane compositions) in a membrane environment (POPC:CHOL = 100:0 and POPC:CHOL = 60:40) at 363 K to assess the stability of the selected TRKB TM dimers, and to obtain relaxed structures. The temperature was chosen based on an objective to foster sampling and to find the thermally most stable dimer structures. These exploratory simulations revealed that the only stable dimer model is centrally-coupled (A439-G443) in a cross-like configuration. The simulations of TRKB presented in this study were all started from conformations sampled from the simulations of these dimers. All membrane-protein systems considered in this study were constructed using CHARMM-GUI (Jo et al., 2017).

##### Drug docking

The drug-binding pocket in the TRKB TM helix dimer was characterized by locally docking FLX to the TRKB TM dimers using RosettaScripts (Fleishman et al., 2011). 30 different cross-like dimer conformations generated by 30 independent 1-μs-long MD simulations in a membrane with 20 mol% cholesterol were used as the “receptor” conformations. FLX was separately placed in the vicinity of the four crevices formed at the dimer interface. The decoys from all docking runs (30 conformations × 4 crevices × 10,000 models) were combined, sorted based on interface score, and visually inspected. The best scoring model, where FLX is bound to the crevice facing the extracellular site in a wide-open cross-like dimer conformation, was selected as the basis for further simulations.

##### Atomistic MD simulations for further exploration and refinement of the drug-binding site

One hundred and twenty independent MD simulations (up to 500 ns each, POPC:CHOL = 80:20, 310 K) were initiated by randomly repositioning and reorienting the drug in the vicinity of the aforementioned TRKB binding pocket by perturbing it from its docked mode. The effect of net positive charge on the stability of FLX binding was assessed by protonation of FLX in place (6 repeats × 500 ns).

##### Production simulations

The details of the simulation systems reported in this study are given in Table S1. The preparation of simulation systems and the simulation protocols follows the strategies described above. Systems 1-4 were simulated at 363 K (Table S1) to improve the sampling of the conformational space of the TRKB dimer structure. In these high temperature simulations, the sampling space was confined to dimeric configurations using half-harmonic flat-bottomed restraints. These studies were guided by recent work on proteins in a membrane environment, which has shown simulations at elevated temperature to accurately reproduce the native-state structural equilibria (Ulmschneider et al., 2011, 2014; Wang et al., 2016). The results of the present work are consistent with this conclusion: the protein fold was not disrupted in any of the cases we simulated, and the wild-type TRKB dimers were stable. However, since TRKB dimers including the Y433F mutation were occasionally less stable at the elevated temperature, the half-harmonic flat-bottomed restraint was used to keep the Y433F dimer stable (system 4). Further, the validity of observations for systems 1-4 (Figure 1) were confirmed by additional simulations at 310 K (systems 5-8). The key results and conclusions were consistent: the simulations at 310 K revealed the same overall distributions as those in Figure 1 but indicated that each one of those is comprised of 2-3 peaks that are separated by a free energy barrier.

As to the Y433F mutation, the results discussed in this paper refer to the heterozygous variant. Additional simulations for the homozygous variant (not discussed here) revealed similar behavior.

Systems 9-14 (Table S1) were prepared starting from the stable FLX-bound state characterized in exploratory simulations described above. The binding site mutations were introduced in systems 12-14. Drug residence times were calculated for systems 10, 12, 13, 14.

Simulations of the TRKA TM region (Table S1, systems 15-20) were carried out in the same way as for TRKB.

##### The estimation of the binding affinity of drugs

For FLX, three independent sets of free energy calculations were performed to estimate the free energy of transfer from the gas phase to the aqueous phase to the membrane phase, and to the protein-binding site at 310 K. All calculations employed the free energy perturbation (FEP) method with Hamiltonian replica exchange (Jiang and Roux, 2010) (exchange attempts at every 1 ps between neighboring windows with an even-odd alternating pattern). Soft-core potentials (Beutler et al., 1994) were employed for both Lennard-Jones and electrostatic interactions (α, the power for lambda term, the power of the radial term, and sigma were set to 0.5, 1, 6, and 0.3, respectively). Each free energy calculation employed 24 windows. For the membrane phase and the protein-binding scenario, the length of the simulations per window was 30-50 ns, and for the aqueous phase 20 ns.

Positional and orientational restraints in the harmonic form were employed to improve phase space overlap in the case of protein-binding calculations, except for the fully coupled state (Wang et al., 2006). Half harmonic restraints were similarly employed to keep the decoupled molecules within the membrane slab in the membrane-transfer simulations. The lambda values, as well as the strength and the center of the restraints, were adjusted to improve the phase space overlap and exchange rates between the lambda windows.

The free energies and their statistical errors were estimated with the Multistate Bennett Acceptance Ratio (BAR) method (Shirts and Chodera, 2008) using Alchemical Analysis and the pymbar software (Klimovich et al., 2015). A separate set of calculations in the gas phase was performed to estimate the necessary corrections for decoupling the intracellular interactions. The necessary corrections arising from the added restraints and the decoupled intra-molecular interactions were applied to obtain the final free energy values.

##### The effects of the drugs on the physical properties of lipid membranes

To test the possibility that drug-induced modulation of lipid bilayer properties could indirectly activate or contribute to the activation of TRKB, we performed additional protein-free simulations of POPC-cholesterol bilayers with varying CHOL concentration (0, 20, and 40 mol%) (Systems 21-50). We used drug-to-lipid ratios of 1:20, 1:10, and 1:5.

##### Simulation protocols

All simulations were carried out using GROMACS 2018 (Abraham et al., 2015). The equations of motion were integrated using the leap-frog algorithm with a 2 fs time step. All bonds involving hydrogens were constrained using the LINCS algorithm (Hess et al., 1997). Long-range electrostatic interactions were treated by the smooth particle mesh Ewald scheme (Essmann et al., 1995) with a real-space cutoff of 1.2 nm, a Fourier spacing of 0.12 nm, and a fourth-order interpolation. A Lennard–Jones potential with a force-switch between 1.0 and 1.2 nm was used for van der Waals interactions.

Before the production runs, all protein-membrane systems were equilibrated in stages with the protein heavy atoms kept restrained, at 310 K and 1 atm pressure using the Berendsen thermostat and barostat (Berendsen et al., 1984).

All production simulations (Table S1) were performed in the NpT ensemble except for systems 1-4 and 15-17, which were performed in the NVT ensemble with a half-harmonic flat-bottomed restraint in case of TRKB (force constant 1000 kJ/mol/nm2) to maintain the inter-helical distance between the G443 Cα atoms below 0.45 nm. The temperature was maintained either at 310 K or 363 K (Table S1) using the Nosé-Hoover thermostat (Hoover, 1985; Nosé, 1984). The protein, the membrane, and the solvent (water and 0.15 M KCl) were each coupled to separate heat baths at the temperatures indicated in Table S1 with a time constant of 1.0 ps. The free energy simulations, on the other hand, employed the leap-frog stochastic dynamics integrator maintaining the temperature at 310 K with the inverse friction constant for each group set to 2.0 ps. Pressure was controlled semi-isotropically using the Parrinello–Rahman barostat (Parrinello and Rahman, 1981) with a reference pressure of 1 atm, a time constant of 5 ps, and compressibility of 4.5 × 10^−5^ bar^−1^ on the *xy*-plane (membrane plane).

The simulation time for systems discussed in this work (the exploratory, the production and the free energy simulations) was over 295 μs. Including a large variety of test simulations (∼330 μs) the total simulation time adds up to more than 625 μs.

#### *In vitro* methods

##### Cell viability

The cell viability assay was performed using CellTiterGlo (#G7571; Promega) according to manufacturer’s instructions. Briefly, equal amounts of medium and the mixture of regents A and B were added to the E18 cortical cells (DIV8) cultivated in clear bottom 96-well plates and incubated for 40min. The luminescence was analyzed in a plate reader.

##### Immunoassays

The TRKB:pY (phosphorylated TRKB), TRKB:PLC-γ1, PLC-γ1:pTRKB.Y816 interactions and were determined by ELISA, based on the general method described in literature (Antila et al., 2014; Fred et al., 2019), with minor adjustments. Briefly, white 96-well plates (OptiPlate 96F-HB, Perkin Elmer) were coated with capturing anti-TRKB or PLC-γ1 antibody (1:1000) in carbonate buffer (pH = 9.8) overnight (ON) at 4°C. Following a blocking step with 2%BSA in TBS-T (2 h, RT), samples were incubated ON at 4°C. The incubation with antibody against pTRKB.Y816, PLC-γ1 or pY (1:2000, ON, 4°C) was followed by HRP-conjugated anti-Rb IgG (1:5000, 2h, RT) or HRP-conjugated streptavidin (1:10000, 2h, RT). Finally, the chemiluminescent signal generated by the reaction with ECL was analyzed in a plate reader (Varioskan Flash, Thermo Scientific).

The surface levels of TRKB were also determined by ELISA (Zheng et al., 2008). Briefly, the MG87.TRKB cells or primary cortical cells from rat or mouse embryos, cultivated in clear bottom 96-well plates (ViewPlate 96, Perkin Elmer), were washed with ice-cold PBS and fixed with 100μl of 4% PFA per well. After washing with PBS and blocking with PBS containing 5% nonfat dry milk and 5% BSA, the samples were incubated with primary anti-TRKB antibody (R&D Systems, #AF1494, 1:1000 in blocking buffer) or anti-GluR1 subunit of AMPA receptors (CST, #8850, 1:2000 in blocking buffer) ON at 4°C. Following washing, the samples were incubated with HRP-conjugated anti-goat or anti-rabbit IgG (1:5000 in blocking buffer) for 1h at RT. The cells were washed 4x with 200μl of PBS for 10 min each. Finally, the chemiluminescent signal generated by reaction with ECL was analyzed in a plate reader.

Protein-fragment complementation assay (Remy and Michnick, 2006) (PCA) was measured upon the reconstitution of enzymatic activity of a humanized *Gaussia princeps* luciferase (GLuc) following the direct interaction of the proteins of interest in white 96-well plates. Two complementary fragments of the luciferase reporter protein were fused to the intracellular C terminus domain of TrkB or mutant TrkB(Y433F) to produce the PCA pairs GLuc1C-TRKB.wt/GLuc2C-TRKB.wt and GLuc1C-TRKB.Y433F/GLuc2C-TRKB.Y433F. Alternatively, the GLuc1C-TRKB.wt/GLuc2C-FYN or GLuc1C-TRKB.Y433F/GLuc2C-FYN pairs were used The GLuc2C-FYN construct expresses a lipid-raft enriched fragment of Src-family kinase FYN (Merezhko et al., 2020). The GLuc tag was linked via a GS linker that allows the physiological dynamics of TrkB without interference from the presence of the tag. When two TrkB molecules carrying the complementary GLuc fragments interact, the reporter can refold in its original and active conformation thereby producing bioluminescence in the presence of its substrate native coelenterazine. Neuro2A cells, in 10% (v/v) poly-L-Lysine coated 96 wells (10,000 cells/well) were transfected with the above-mentioned constructs. Cells were treated 48h post-transfection with BDNF (10ng/ml/10min), and luminescence measured as a direct indication of TRKB homodimerization with a plate reader (Varioskan Flash, Thermo Scientific, average of 5 measurements, 0.1 s each) immediately after the injection of the coelenterazine substrate (Nanolight Technology).

The levels of total and phosphorylated TRKB at Y515 or Y816 in MG87.TRKB cells, challenged with BDNF, as well as the levels of PLCg1 and pTRKB.Y816 were measured by western-blotting (Rantamäki et al., 2007).

For the analysis of TRKB migration to lipid rafts, the samples from transfected N2A cells to express GFP-tagged TRKB.wt or TRKB.Y433F, challenged with BDNF, were processed to isolate detergent-resistant membrane (DRM) fractions in sucrose gradient (McGuinn and Mahoney, 2014). N2A cells were seeded at a density of 2.5 million per plate on 10 cm plates and transfected after 24 hours with either wild-type GFP-tagged full length TRKB or the GFP-tagged Y433F TRKB mutant. 48 hours after plating, cells were washed with ice cold 1x PBS and scraped in extraction buffer (25 mM Tris-HCl pH 8, 150 mM NaCl, 5 mM EDTA) with the addition of 0.5% v/v Lubrol (Serva) and a cocktail of protease and phosphatase inhibitors (Sigma). Cellular membranes were mechanically broken by passing the cell suspension through a 23G needle five times. Protein concentration was measured for each sample and equal amounts of proteins were transferred to Eppendorf tubes and mixed with sucrose in the extraction buffer to a final concentration of 72%. The samples were then transferred to the bottom of Beckman 2.2 mL ultracentrifuge tubes and carefully covered with equal volumes of 35% sucrose and 5% sucrose in the extraction buffer.

The samples were centrifuged at 52000 x g for 18 hours at +4°C with a TLS-55 rotor in a Beckman Coulter XP Optima ultracentrifuge. Finally, 12 fractions per sample, collected from the top of the tube, were transferred to clean tubes, sonicated for 10 minutes in 0.25% SDS and prepared for western blotting, where the levels of GFP-tagged TRKB and flotillin-2 were analyzed.

##### Ligand binding assays

The cell-free assays (binding assays) were performed in white 96-well plates, based on the protocol used for western-blotting or ELISA (Baeza-Raja et al., 2016; Das et al., 2015). The plates were precoated with anti-GFP, anti-FLAG, anti-TRK or anti-TRKB antibody (1:1000) in carbonate buffer (pH 9.8), ON at 4°C. Following blocking with 3% BSA in PBS buffer (2 h at RT), 120 ug of total protein from each sample (of lysates from HEK293T cells transfected to overexpress GFP-TRKB.wt or GFP-TRKB.Y433F) were added and incubated overnight at 4°C under agitation. As controls, lysate from MG87 cells expressing TRKA were compared with cells expressing TRKB, and HEK293T cells expressing GFP-TRKB were compared with non-transfected cells, see Figure S4. The plates were then washed 3x with PBS buffer, and the biotinylated fluoxetine or RR-HNK (0-100μM), or a mixture of biotinylated (1μM of fluoxetine or R,R-HNK) and non-biotinylated compounds (0-10μM), was added for 1h at RT. The amino-biotinylation of fluoxetine and RR-HNK was performed using a commercial kit (EZ-Link NHS-PEG4 Biotinylation Kit, #21455, Thermo Scientific) and the reaction monitored by mass spectrometry (Tikka et al., 2016). The luminescence was determined via HRP-conjugated streptavidin (1:10000, 1h, RT) activity reaction with ECL by a plate reader. The luminescence signal from blank wells (containing all the reagents but the sample lystates, substituted by the blocking buffer) was used as background. The specific signal was then calculated by subtracting the values of blank wells from the values of the samples with matched concentration of the biotinylated ligand. For Figure 2E, the precipitated TRKB.wt or Y433F was incubated with tritiated imipramine (0-30μM) and the radioactive emission of the ligand was determined by scintillation (OptiPhase Supermix cocktail: #1200-439; MicroBeta2, Perkin-Elmer). The scintillation signal from blank wells was used as background, and the specific signal was then calculated by subtracting the values of blank wells from the values of the samples with matched concentration of the radioactive ligand.

Microscale Thermophoresis - MST - experiments were performed using Monolith NT.115 (Blue/Red) instrument (NanoTemper Technologies GmbH, Germany). The cell lysates of HEK293T cells expressing GFP-TRKB were used as a source of fluorescently labeled TRKB. HEK293T cells were transfected with GFP-fused TRKB as described and lysed 24h after transfection. To evaluate binding of fluoxetine to TRKB, cell lysates were diluted 1.5x times with MST buffer (10mM Na-phosphate buffer, pH 7.4, 1mM MgCl2, 3mM KCl, 150mM NaCl, 0.05% Tween-20) to provide optimal level of fluorescence. The lysates of non-transfected HEK293T cells were used to evaluate background fluorescence, which appeared to be undetectable in current MST set up. Titration series of fluoxetine (0-100μM) were incubated with diluted cell lysates. The measurements were done in premium coated capillaries (NanoTemper Technologies GmbH, MO-K025) using LED source with 470nm and 50% infrared-laser power at 25°C. Hill model was used to evaluate binding affinity, each data point represents mean ΔFnorm values from four independent experiments. The bound fraction of the GFP-TRKB was determined and the fitting was performed using the Hill model method incorporated by the MO Affinity Analysis v2.3 software (NanoTemper Technologies).

##### In situ proximity ligation assay (PLA)

Proximity ligation assay was done with Duolink *In Situ* Red Starter Kit Mouse/Goat (#DUO92103, Sigma-Aldrich), following manufacturer’s instructions. Briefly, N2A cells (grown in 13 mm glass coverslips, in DMEM 10% FBS) were co-transfected by lipofectamine 2000 (ThermoFisher) to express TRKB and farnesylated EGFP and, 24h later, were treated with biotinylated fluoxetine (10 μM/15min). Cells were fixed with PFA 4% for 15 min, washed, and incubated in Duolink blocking buffer for 1h at 37°C. Then, the coverslips were incubated with streptavidin-conjugated HRP (1:5000 in Duolink antibody diluent) for 45 min at room temperature. After washing twice in Duolink washing buffer A, samples were incubated overnight at 4°C with a mix of goat anti-TRKB (1:1000, #AF1494, R&D System) and mouse anti-HRP (1:1000, #200-3138-0100, Rockland Immunochemicals) diluted in Duolink antibody diluent. After washing in Duolink washing buffer A, samples were incubated for 1h at 37°C with a mix of minus and plus probes diluted 1:5 in Duolink antibody diluent. Then, ligation of probes with connector oligo was done by washing the samples in washing buffer A, and incubating for 30min at 37°C with ligase (25 U/ml) diluted in the Duolink ligation buffer. Amplification was done by washing the samples in the washing buffer A, and incubating for 100min at 37°C with polymerase (125 U/ml) diluted in the amplification buffer. Coverslips were washed in buffer B, then in buffer B diluted 1:100 in milliQ water, and mounted in Duolink *in situ* mounting medium with DAPI. All incubation steps were done in a humidified chamber.

Images were acquired in z stacks (at least 15 slices, 1.5μm each) with a Zeiss LSM 710 confocal microscope (objective alpha Plan-Apochromat 63x/1.46 oil Korr M27, with 3x zoom).

##### Fluorescence recovery after photobleaching

For the fluorescence recovery after photobleaching (FRAP) experiments, E18 rat embryo hippocampal cells were plated onto glass coverslips coated with poly-L-lysine (Sigma-Aldrich) in 4-well cell culture plates at a density of 200.000 cells/well. The cultures were incubated at +37°C in serum-free Neurobasal medium (supplemented with 2% B27, 1% L-glutamine and 1% ampicillin) for approximately 2 weeks (DIV 14-16), renewing half of the growth media weekly. Then, they were transfected with Lipofectamine 2000 (Invitrogen) in order to overexpress GFP-tagged TRKB constructs (TRKB.WT or TRKB.Y433F). The cultures were incubated with the transfection mix (per well: 50ul neurobasal medium, 1ul Lipofectamine 2000, 1ug plasmid DNA) in serum-free media for 1.5 hours, followed by 3x medium washes and returned to the original growth medium after that. The cells were left at +37°C o/n to overexpress the constructs (Koskinen et al., 2012). Next day, FRAP was performed with a confocal microscope (Zeiss LSM 710, Carl Zeiss AG) adapted for live cell imaging with a chamber set at +37°C and 5%CO2 (Zeiss Temp Module system, Carl Zeiss AG). Coverslips with the cultured hippocampal neurons were carefully transferred to 35mm Petri dishes with warm HBSS medium without phenol red or serum to avoid sources of background fluorescence. After a 10 minutes adaptation period in the microscope chamber to avoid focus drift caused by temperature fluctuations of the stage and objective, dendritic shafts or spines (spine head diameter = ∼1um) were localized with a 63x/0.90 NA dipping water objective. Picture format was 512x512 pixels. The nominal speed to capture images was set to 9 (pixel dwell 3.15 μseconds, taking approximately 0.5 s to finish a scan), and the pinhole aperture was set to completely open to obtain a strong fluorescent signal (Koskinen et al., 2012; Zheng et al., 2011). Once a region of interest (ROI) was selected with a circle that contained the whole spine, it was bleached to establish the FRAP baseline. In the prebleaching phase, three frames were taken for the purpose of normalization, setting the first frame as 100% FRAP start value. The bleaching phase consisted of a very short cycle (1-5 bleach iterations, ∼0.01–0.5 s) of bleaching to decrease signal of the ROI to 50%. The postbleaching phase lasted for 2 minutes (with a time resolution of 2 s/frame), until a plateau was reached and the shape of the recovery curve was clear. Next, fluoxetine (1μM), ketamine (10μM) or BDNF (20ng/ml) was added to the medium to reach a concentration of and was incubated for 15 minutes. After FRAP establishing baseline, bleaching was performed in different dendritic shafts or spines, and the following recording lasted again for 2 minutes (time resolution of 2 s/frame). Once added to the medium, the drug was present for the rest of the FRAP recording. Finally, for the FRAP image analysis we used the native Zeiss LSM 710 Zen software. The intensity value of the bleached ROI is normalized to a second neighboring unbleached dendritic ROI of the same area and similar initial intensity in order to compensate for the photobleaching generated in the acquisition of the images. That intensity value is then converted to % by dividing the intensity values of individual frames by the start value of the first frame (t0, 100%). The mean values from the different experimental groups were then plotted to generate the recovery curve (González-González et al., 2012; Koskinen et al., 2012).

##### Immunostaining

MG87.TRKB or primary cultured cortical cells were fixed with 4% paraformaldehyde in PBS, and unspecific binding sites were blocked with a blocking buffer (5% normal donkey serum, 1% Bovine serum albumin, 0.1% gelatin, 0.1% Triton X-100, 0.05% Tween-20 in PBS). The MG87.TRKB cells were stained with anti-GFP, while primary cultures were incubated with anti-actin. The fluorescence was obtained following incubation with secondary antibodies. The coverslips were mounted with Dako Fluorescence Mounting Medium (#S3023). Images were acquired with Zeiss LSM710 confocal microscope (63x oil objective).

For Sholl analysis of cultured hippocampal cells, confocal images were processed using FIJI ImageJ (NIH software), and the number of branches in the 80-μm range from the cell soma were counted using FIJI plugin.

##### Super resolution microscopy (dSTORM/TIRF)

For direct stochastic optical reconstruction microscopy (dSTORM) MG87.TRKB cells were grown on cell view 35mm dishes with glass bottom (Greiner) coated with poly-L-lysine (Sigma). Cells were transfected to overexpress TRKB.wt or TRKB.Y433F, and 24h later treated with BDNF (10 ng/ml), fluoxetine (10 μM), cholesterol (20 μM) or ketamine (10 μM) for 15 minutes and fixed as for regular immunofluorescence imaging. Briefly, cells were fixed 24 hours post transfection with 4% PFA for 20 minutes, washed three times for 5 min with 1x PBS and incubated in blocking buffer (1% BSA, 0.1% gelatin, 5% goat serum, 0.1% Triton X-100 and 0.05% Tween-20 in PBS; all from Sigma) for 1 hour. The primary antibody against GFP (Abcam, #ab290) was diluted 1:100 and incubated overnight at +4°C, while the secondary antibody Alexa Fluor-conjugated goat-anti-rabbit 647 (Invitrogen) was diluted 1:500 and incubated at room temperature for 1 hour. The dishes were stored in +4°C in 1x PBS no longer than 48 hours before imaging.

During imaging, cells were maintained in blinking buffer (10% glucose, 0.07% cysteamine, 0.75 mg/ml glucose oxidase and 0.04 mg/ml catalase in 0.1 M Tris buffer, pH 7.8-8; all reagents from Sigma-Aldrich), which was freshly prepared and changed every hour. Imaging was performed in TIRF mode (TIRF angle 89.3 calibrated with ring-TIRF at least twice per sample) to specifically image the fluorophores at the plasma membrane. A GE DeltaVision OMX SR system (GE Healthcare) equipped with an Apo N 60x/1.49 oil objective and a sCMOS camera was used to acquire the images. The 640 nm diode laser was used at 100% of power to excite the Alexa 647 fluorophores while the 405 nm laser was adjusted until approximately 50% of power in order to maintain the blinking throughout the imaging session. For each cell, 35000 frames were acquired, and the localization map of single fluorophores was reconstructed with SoftWoRx 7.0 software (GE Healthcare). For the localization map, the first 1000 frames approximately were discarded and the reconstruction parameters were maintained identical for all the samples.

Reconstruction parameters: For the localization of all the blinking events in the dataset, the point spread function size factor was set at 1.550 with a local maximum factor of 0.05. Drift correction was adjusted dividing the frames into 20 groups. Tracking of the fluorophores was performed with a maximum localization precision of 100 nm without fiducial markers. The final localization map of the fluorophores identified was built with a reconstruction pixel size of 10 nm and localization precision set at 5 and 100 nm respectively for the minimum and the maximum, and a fluorophore persistence threshold set at 3 frames.

The analysis of the reconstructed images was performed in Fiji. Depending on the size of picture, 5 or 10 representative regions of interest (ROIs) of 2x1 μm were blindly chosen per cell and used for particle analysis of the TRKB-positive clusters. The images acquired were from three independent experiments (Jacobson et al., 2007; Pralle et al., 2000; Salo et al., 2016).

#### *In vivo* methods

##### Electrophysiology

*In vivo electrophysiology.* Intrahippocampal BDNF infusion was performed as described previously (Messaoudi et al., 1998). The experiments were carried out on 20 adult male Sprague-Dawley rats (15 weeks old, Janvier, France). Rats were housed in the animal facility for 1-week prior to the start of drug administration. Pravastatin was administered in drinking water at a dose of 10mg/kg/day (0.08mg/l solution) for 15-17 days calculated on the basis of daily body weight.

For electrophysiological experiments, rats were anesthetized with an intraperitoneal injection of urethane (U2500, Sigma-Aldrich) 1.5 g/kg body weight, positioned in a stereotaxic frame. The body temperature was constantly monitored and maintained at 37°C with an electric heating pad. Holes were drilled in the skull and a concentric bipolar stimulating electrode (Tip separation 500micrometers; SNEX 100; Rhodes Medical Instruments, Woodland Hills, CA) was positioned in the angular bundle for stimulation of the medial perforant path (7.9 mm posterior to bregma, 4.2 mm lateral to the midline, and 2.5 mm below the dura.

A Teflon-coated tungsten wire recording electrode (outer diameter of 0.075 mm; A-M Systems #7960) was glued to the infusion cannula (30 gauge). The electrode was then cut so that it extended 800 μm from the end of the cannula. The recording electrode was placed in the dentate hilus (3.9 mm posterior to bregma, 2.2 mm lateral, and 2.8–3.1 mm below the dura). The recording electrode was slowly lowered into the brain with 0.1 mm increment while monitoring response waveform evoked at 400 μA with biphasic rectangular test pulse of 0.033 Hz (pulse-width 0.15 ms). The tip of the infusion cannula was located in the deep stratum lacunosum-moleculare of field CA1, 800 μm above the hilar recording site and 300-400 μm above the medial perforant synapse. The infusion cannula was connected via a polyethylene (PE50) tube to a 10μl Hamilton syringe and infusion pump.

Responses were allowed to stabilize for 1 hour at a stimulation intensity that produced a population spike 30% of maximum. After baseline recording for 20 min, infusion of 2μl of 1 ug/ul BDNF over 30 min at a rate of 0.067μl/min. Evoked responses were recorded for 120 min post infusion. Recorded field potentials were amplified, filtered (0.1 Hz to 10 kHz), and digitized (25 kHz). Electrophysiological data was analyzed using Datawave Technologies Software company. Changes in the fEPSP slope were expressed in percent of baseline. After recordings rats were decapitated and dentate gyri were dissected and immediately frozen on dry ice and stored at −80°C until use.

*Ex vivo electrophysiology.* Extracellular field potentials (fEPSP) were recorded in acute slices of adult mouse hippocampus (Popova et al., 2017). Adult male and female mice (18-22 weeks old) were deeply anaesthetized with isoflurane, the brains were dissected and immersed in ice-cold dissection solution containing (in mM): 124 NaCl, 3 KCl, 1.25 NaH2PO4, 1 MgSO4, 26 NaHCO3, 15 D-glucose, 9 MgSO4 and 0.5 CaCl2. The cerebellum and anterior part of the brain were removed and horizontal 350 μm brain slices of the hippocampus were cut on a vibratome. For recovery, the slices were incubated for 30 min at 31-32°C in artificial cerebrospinal fluid (ACSF) containing (in mM): 124 NaCl, 3 KCl, 1.25 NaH2PO4, 1 MgSO4, 26 NaHCO3, 15 D-glucose, and 2 CaCl2 and bubbled with 5% CO2/95% O2.

Field excitatory postsynaptic currents (EPSPs) were recorded in an interface chamber using ACSF-filled glass microelectrodes (2-4 MΩ) positioned in the stratum radiatum of the CA1 region and amplified an Axopatch 200B amplifier. Electric stimulation (100 μsec duration) was delivered with a bipolar concentric stimulation electrode placed at the Schaffer collateral. Baseline synaptic responses were evoked every 20 s with a stimulation intensity that yielded a half-maximum response. After obtaining a 15 min stable baseline theta burst stimulation (TBS: 10 bursts of four pulses at 100 Hz, with an interburst interval of 200 msec) or tetanic stimulation (200ms pulse interval; 100 pulses; 0.1ms pulse duration) was delivered and field potentials were recorded for 45 min.

Input/output (I/O) curves were constructed using gradually increased stimulation intensities of 5, 10, 20, 30, 40 and 50 mV until the fEPSP reached plateau or visible population spike was seen.

To examine short term plasticity, we performed paired-pulse-facilitation (PPF) experiments using interpulse intervals (IPIs) of 20, 50, 100, and 200 ms and stimulation intensity evoking half-maximal fEPSP slopes were used. WinLTP (0.95b or 0.96, https://www.winltp.com) was used for data acquisition.

##### Ocular dominance plasticity

The “transparent skull” preparation was executed as described in literature (Steinzeig et al., 2017). Briefly, adult female mice (20-22 weeks old) were anesthetized via i.p. injection with a mixture containing: 0.05 mg/kg fentanyl (Hameln, Germany); 5 mg/kg midazolam (Hameln, Germany); 0.5 mg/kg medetomidine (Orion Pharma, Finland); diluted in saline (B Braun, Germany). The animals’ eyes were covered with protective-gel (Alcon, UK). The animal’s head was shaved and disinfected with ethanol and Betadine. The mouse head was then fixed on the stereotaxic frame and the temperature was maintained at 37°C. A mixture of lidocaine and adrenaline (Orion Pharma, Finland) was applied locally on the head and the skin was cut with spring scissors. The incision area was restricted 2 mm above the bregma and caudal edge of intraparietal bone. The scalp was removed and a focused air stream and a cotton swab were used to clean and stop bleeding. The periosteum was gently scratched away from the skull with an eye scalpel and the temporal muscles were pushed aside, in order to expose enough space around the area of interest to attach the metal holder. When the skull was smooth, white and dry, fat was removed from the skull rapidly by passing on it a cotton swab soaked in acetone and the air current was used to avoid its penetration into the bone. Then, with a metal stick, a thin layer of cyanoacrylate glue (Loctite 401, Henkel, Germany) was applied to the surface of the skull, in order to make the skull transparent. After waiting 10 minutes for the glue to dry, a first layer of acryl was applied on the skull surface with a brush. Acryl was used to prolong the life of the transparency and was prepared by stirring acrylic powder (EUBECOS, Germany) with methacrylate liquid (Densply, Germany) until a nail-polish consistency was reached. After 30 minutes, a second layer of acryl was applied and was left to dry overnight. Finally, the animals were injected s.c. with 5 mg/kg Carprofen (ScanVet, Nord Ireland) for postoperative analgesia and i.p. with a wake-up composed by: 1.2 mg/kg Naloxone (Orpha-Devel Handels und Vertriebs GmbH, Austria), opioid receptor antagonist; 0.5 mg/kg Flumazenil (Hameln, Germany), GABA-A receptor antagonist; 2.5 mg/kg Atipamezole (Vet Medic animal Health Oy, Finland), adrenergic receptor antagonist; diluted in saline (B Braun, Germany). The next day, isoflurane at 4% was used for a couple of minutes to induce the anesthesia and then it was reduced at 2% for the procedure. The acryl layer was polished only on the area of interest, using a hand drill with a polishing bit. A metal head holder was first glued on the skull, carefully keeping the area of interest at the center of the holder, and then fixed with a mixture of cyanoacrylate glue and dental cement (Densply, Germany). Finally, transparent nail polished (#72180, Electron Microscopy Sciences) was applied inside the metal holder above the area of interest.

*Monocular deprivation.* -Isoflurane at 4% was used for a couple of minutes to induce the anesthesia and then it was maintained at 2% until the end of the procedure. A drop of antibiotic eye gel (Isothal Vet 1%, Dechra, Canada) was applied on the left eye, the eyelashes were cut and the eye was sutured shut with 3 mattress sutures. At the end, antibiotic ointment (Oftan Dexa-Chlora, Anten, Finland) was applied on the sutured eye and Carprofen (5 mg/kg) was injected s.c. for postoperative analgesia. The monocular deprivation lasted 8 days and during that period, all animals were checked daily and resutured when showed a sign of the thread loss to prevent reopening of the eyes.

*Optical imaging.* The saturation of haemoglobin in the primary visual cortex of the right hemisphere was measured to assess cortical activity (Cang et al., 2005; Kalatsky and Stryker, 2003). The animals were anesthetized with 1.8% isoflurane with a 1:2 mixture of O2:air for 15 minutes and then maintained at 1.2% isoflurane for at least 10 minutes before starting the imaging session. Two sessions of imaging were performed: one before the beginning of the treatment administration (IOS I) and one on the 8th day after monocular deprivation (IOS II).

*Optical Imaging Apparatus.* The animals were kept on a heating pad located in front of and within 25 cm from the stimulus monitor. The head holder was firmly fixed and the animal’s nose was aligned to the midline of the stimulus monitor. The visual stimulus was a 2° wide horizontal bar moving upward with a temporal frequency of 0.125 Hz and a spatial frequency of 1/80 degree, displayed in the central part of a high refresh rate monitor (−15 to 5 degree azimuth, relative to the animal visual field) in order to preferentially stimulate the binocular part of the visual field. First, a green light (540 ± 20 nm) was used to illuminate the skull surface in order to find the region of interest and acquire a map of the surface vascular pattern, then the camera was focused 600 μm below the pial surface and the green light switched with a red light (625 ± 10 nm), to record the intrinsic signal. A longpass red filter (590 nm) was put between the skull surface and the camera to avoid that light from the stimulus monitor could affect the intrinsic signal acquisition. When the signal from one eye was being recorded, the other eye was protected with viscotears and covered with a patch. The continuous-periodic stimulation was synchronized with a continuous frame acquisition, frames were collected independently for each eye at a rate of 30 fps for 5 minutes and stored as a 512 × 512 pixel image, after spatial binning of the camera images.

*Data analysis.* An analysis software package designed by Kalatsky et al. was used to perform Fourier decomposition on the cortical maps, that allowed to extract the intrinsic signal from the biological noise (Kalatsky and Stryker, 2003). The intrinsic response was presented as fractional changes in skull surface reflectance x 104 and its magnitude was used to calculate the activation of the visual cortex in the right hemisphere due to ipsilateral or contralateral eye stimulation. A low-pass filter (uniform kernel of 5 × 5 pixel) was applied to the ipsilateral magnitude map to smoothen it and then, in the resulting map, the 30% of the peak response amplitude was set as threshold in order to eliminate background noise and to define the area that produced the strongest response to the ipsilateral eye. This smoothened and thresholded map was used as a mask to select the binocularly responsive region of interest within the visual cortex. After obtaining cortical maps for both contralateral (C) and ipsilateral (I) eyes and computing Ocular Dominance score as (C−I)/(C+I), finally, the Optical Dominance Index (ODI) was calculated as the mean of the OD score for all responsive pixels (Cang et al., 2005; Greifzu et al., 2014). The ODI values are contained in an interval going from −1 to +1: positive values indicate a contralateral bias, negative ones indicate ipsilateral bias and ODI values of 0 indicate that ipsilateral and contralateral eyes are equally strong (Steinzeig et al., 2017).

##### Behavioral analysis

Object-location memory (OLM): this test was performed in a square arena (28cm side) with opaque walls containing cues (black stripes or spots). The adult male and female mice (18-22 weeks old) were placed for 3 consecutive days (15min per session) in the arena with two identical objects (table tennis balls glued to caps of 50ml Falcon tubes) in the same position throughout the sessions (pretest). At the test session one of the objects is moved to a different position and the number of visits (counted as sniffing or interacting with the object) to the old (A) or newly located (A’) object was determined by an observer bling to the conditions (Ampuero et al., 2013; Dere et al., 2007).

Contextual fear conditioning: this test was modified from previous studies (Karpova et al., 2011). Briefly, adult male and female mice (18-22 weeks old) were conditioned to 5 scrambled foot shocks (0.6mA/2 s) during the 8 min session (arena: 23 × 23 × 35cm) under constant illumination (100 lux). During the extinction trials, the animals were exposed to the same context where the shocks were delivered and the time spent in freezing during the 8 min session was automatically determined by the software (TSE Systems, Germany).

Forced swimming test: animals (adult male and female mice, 18-22 weeks old) were placed in 5l glass beaker cylinders (19cm diameter, with 20cm water column) for 6min. The immobility was assessed in the last 4min of the session (Diniz et al., 2018). The water was changed between each test. After swimming, animals were towel-dried and kept in a warmed cage before returning to their home cages. Test was videotaped and analyzed by a trained observer blind to treatment.

##### Incorporation of BrdU

Animals: both males and females were used in this experiment. The Bromodeoxyuridine (BrdU) (Sigma) was administered intraperitoneally at the dose of 75 mg/kg four times every 2 hr to reach 300 mg/kg total for each animal. The injection procedure was carried out at the start of the treatment (fluoxetine 15mg/kg/day in the drinking water for 3 weeks, solution at 0.1mg/l). On day 21 of the drug treatment, the animals were euthanized by CO2, brains were quickly removed, followed by hippocampus dissection on ice, instantly frozen on dry ice and stored at −80°C until further use.

A semiquantitative dot-blot method was performed as previously described with minor modifications (Wu and Castrén, 2009). To isolate the DNA of the hippocampus samples, DNeasy® Blood and Tissue Kit (QIAGEN, Germany) was used. The extraction procedure was as per the manufacturer’s instruction. The DNA purity was assessed by the spectrophotometer NanoDrop 2000C (ThermoFisher Scientific, USA). DNA was incubated with 1 volume of 4N NaOH solution for 30 min at room temperature to render it as single stranded and immediately kept on ice to prevent reannealing. The DNA solution was neutralized by an equal volume of 1M Tris-HCl (pH 6.8). The single-stranded neutralized DNA (1ug) was dot-blotted onto a nylon transfer membrane (Schlleicher and Schuell, Keene, NH) with a dot-blot apparatus (Minifold, Schlleicher and Schuell) under vacuum and the DNA was fixed by ultraviolet cross-linker (1200 μJ × 100, Stratagene, La Jolla, CA). These membranes were processed as mentioned in the previous publication. The mouse anti BrdU monoclonal antibody (1:1000, B2531, Sigma) was used as the primary antibody and anti-mouse horseradish peroxidase (HRP) (Bio-Rad, USA) as the secondary antibody. The Pierce ECLplus kit (Thermo Fisher scientific, USA) was used as a chemiluminescent method to develop the membrane. The membranes were scanned by imaging using a Fuji LAS-3000 Camera (Tamro Medlabs, Finland) and the densitometry analysis was performed by ImageJ Software.

##### Quantification of brain fluoxetine levels

The brain samples were homogenized in 200 μL of 100% methanol (LiChrosolv, Merck, Darmstadt, Germany) with an ultrasound sonicator (GM35-400, Rinco Ultrasonic, Switzerland) continuously for at least 30 s. The homogenates were centrifuged at 20800 g for 35 min at 4°C. The supernatant was then removed to 0.5 mL Vivaspin filter concentrators (10,000 MWCO PES; Vivascience AG, Hannover, Germany) and centrifuged at 8600 x g at 4°C for 35 min.

The HPLC system for determination of the concentration of fluoxetine consisted of a solvent delivery pump (Jasco model PU-1580 HPLC Pump, Jasco International Co, Japan) connected to an online degasser (Jasco 3-Line Degasser, DG-980-50) and a ternary gradient unit (Jasco LG-1580-02), an analytical column (Kinetex C-18 5 μm, 4.60 × 50 mm, Phemomenex Inc) protected by a 0.5-mm inlet filter and thermostated by a column heater (CROCO-CIL, Cluzeau Info-Labo, France), and a fluorescence detector (Jasco Intelligent Fluorescence Detector model FP-1520). The wavelengths of the fluorescence detector were set to 290 (excitation) and 230 (emission), which have been found to be optimized for fluoxetine (Raggi et al., 1998). The mobile phase consisted of 0.1 M NaH2PO4 buffer (Merck, D), pH 2.7 (adjusted with phosphoric acid), 35% (v/v) acetonitrile (LiChrosolv, Merck, Darmstadt, Germany), and the flow-rate was 1.0 ml/min. Ten microliters of the filtrate was injected onto the column with a refrigerated autoinjector (Shimadzu NexeraX2 SIL-30AC, Shimadzu Corp, Japan). The chromatograms were processed by AZUR chromatography data system software (Cromatek, Essex, UK). The results can be found in Table S2.

### Quantification and statistical analysis

The data in the present study was analyzed by Student’s t test (two-tailed, paired or independent); one- or two-way ANOVA were also applied, followed by Fisher’s LSD *post hoc* test using GraphPad Prism (https://www.graphpad.com/) v6.01; or multivariate ANOVA using JASP (https://jasp-stats.org/) v.0.14.1. The *F* and *p* values are indicated in the figure legends. All experimental data used in the present study are available in FigShare under CC-BY license (https://doi.org/10.6084/m9.figshare.12698012).

## Supplemental information

Table S1. Simulated systems discussed in this study, related to Figures 1G–1J and 3 The table lists the variant of TRKB dimers, mole percentage of cholesterol (*ρ* CHOL ), the number of POPC ( *N* POPC ), cholesterol ( *N* CHOL ), and drug ( *N* DRUG ) molecules, temperature ( *T* ), the number of simulation repeats ( *N* sim ), and the simulation length per repeat (*t* sim ). “WT”, “Y433F.het”, “V437A.hom”, “S440A.hom” refer in respective order to the wild-type, heterozygous Y433F, homozygous V437A, and homozygous S440A variants of TRKB TM dimer (residues 427-459) and TRKA TM dimer (residues 410-443). FLX: fluoxetine, SKE: S-ketamine “Protein-free” refers to the systems without the protein in the membrane.

Table S2. Levels of fluoxetine (15 mg/kg in the drinking water for 21 days) in the prefrontal cortex of mice, related to Figures 5, 6, and S6.
