# Supplementary material for: Antidepressant drugs act by directly binding to TRKB neurotrophin receptors
Source: Cell. 2021 Mar 4;184(5):1299–1313.e19. doi: 10.1016/j.cell.2021.01.034 (PMC7938888; doi:10.1016/j.cell.2021.01.034)
Supplement: Table S1. Simulated systems discussed in this study, related to Figures 1G–1J and 3 — The table lists the variant of TRKB dimers, mole percentage of cholesterol (ρ CHOL ), the number of POPC ( N POPC ), cholesterol ( N CHOL ), and drug ( N DRUG ) molecules, temperature ( T ), the number of simulation repeats ( N sim ), and the simulation length per repeat (t sim ). “WT”, “Y433F.het”, “V437A.hom”, “S440A.hom” refer in respective order to the wild-type, heterozygous Y433F, homozygous V437A, and homozygous S440A variants of TRKB TM dimer (residues 427-459) and TRKA TM dimer (residues 410-443). FLX: fluoxetine, SKE: S-ketamine “Protein-free” refers to the systems without the protein in the membrane. [file mmc1.pdf]

Cell, Volume *184*

Supplemental information

Antidepressant drugs act by directly binding to TRKB neurotrophin receptors

Plinio C. Casarotto, Mykhailo Girych, Senem M. Fred, Vera Kovaleva, Rafael Moliner, Giray Enkavi, Caroline Biojone, Cecilia Cannarozzo, Madhusmita Pryiadrashini Sahu, Katja Kaurinkoski, Cecilia A. Brunello, Anna Steinzeig, Frederike Winkel, Sudarshan Patil, Stefan Vestring, Tsvetan Serchov, Cassiano R.A.F. Diniz, Liina Laukkanen, Iseline Cardon, Hanna Antila, Tomasz Rog, Timo Petteri Piepponen, Clive R. Bramham, Claus Normann, Sari E. Lauri, Mart Saarma, Ilpo Vattulainen, and Eero Castrén

Table S1. Simulated systems discussed in this study. The table lists the variant of TRKB dimers, mole percentage of cholesterol (ρ_{CHOL}), the number of POPC (N_{POPC}), cholesterol (N_{CHOL}), and drug (N_{DRUG}) molecules, temperature (T), the number of simulation repeats (N_{sim}), and the simulation length per repeat (t_{sim}). "WT", "Y433F.het", "V437A.hom", "S440A.hom" refer in respective order to the wild-type, heterozygous Y433F, homozygous V437A, and homozygous S440A variants of TRKB TM dimer (residues 427-459) and TRKA TM dimer (residues 410-443). FLX: fluoxetine, SKE: S-ketamine "Protein-free" refers to the systems without the protein in the membrane. Related to Fig. 1G-J and Fig. 3.

System name	Protein variant	Drug type	ρ_{CHOL} (mol%)	N_{POPC}	N_{CHOL}	N_{DRUG}	T (K)	N_{sim}	t_{sim} (μs)
System 1[†]	WT TRKB		0	128	0		363	10	1
System 2[†]	WT TRKB		20	112	28		363	10	1
System 3[†]	WT TRKB		40	90	60		363	10	1
System 4[†]	Y433F.het TRKB		20	112	28		363	10	1
System 5	WT TRKB		0	128	0		310	10	1
System 6	WT TRKB		20	112	28		310	10	1
System 7	WT TRKB		40	90	60		310	10	1
System 8	Y433F.het TRKB		20	112	28		310	10	1
System 9	WT TRKB	FLX	0	128	0	1	310	10	1
System 10	WT TRKB	FLX	20	112	28	1	310	10	1
System 11	WT TRKB	FLX	40	90	60	1	310	10	1
System 12	Y433F.het TRKB	FLX	20	112	28	1	310	20	1
System 13	V437A.hom TRKB	FLX	20	112	28	1	310	10	1
System 14	S440A.hom TRKB	FLX	20	112	28	1	310	10	1
System 15[†]	WT TRKA		0	128	0		363	10	1
System 16[†]	WT TRKA		20	112	28		363	10	1
System 17[†]	WT TRKA		40	90	60		363	10	1
System 18	WT TRKA		0	128	0		310	10	1
System 19	WT TRKA		20	112	28		310	10	1
System 20	WT TRKA		40	90	60		310	10	1
System 21	protein-free		0	200	0		310	1	0.5
System 22	protein-free		20	160	40		310	1	0.5
System 23	protein-free		40	120	80		310	1	0.5
System 24	protein-free	FLX	0	200	0	10	310	1	0.5
System 25	protein-free	FLX	20	160	40	10	310	1	0.5
System 26	protein-free	FLX	40	120	80	10	310	1	0.5
System 27	protein-free	FLX	0	200	0	20	310	1	0.5
System 28	protein-free	FLX	20	160	40	20	310	1	0.5
System 29	protein-free	FLX	40	120	80	20	310	1	0.5
System 30	protein-free	FLX	0	200	0	40	310	1	0.5
System 31	protein-free	FLX	20	160	40	40	310	1	0.5
System 32	protein-free	FLX	40	120	80	40	310	1	0.5
System 33	protein-free	SKE	0	200	0	10	310	1	0.5
System 34	protein-free	SKE	20	160	40	10	310	1	0.5
System 35	protein-free	SKE	40	120	80	10	310	1	0.5
System 36	protein-free	SKE	0	200	0	20	310	1	0.5
System 37	protein-free	SKE	20	160	40	20	310	1	0.5
System 38	protein-free	SKE	40	120	80	20	310	1	0.5
System 39	protein-free	SKE	0	200	0	40	310	1	0.5
System 40	protein-free	SKE	20	160	40	40	310	1	0.5
System 41	protein-free	SKE	40	120	80	40	310	1	0.5

System 42	protein-free	R,R-HNK	0	200	0	10	310	1	0.5
System 43	protein-free	R,R-HNK	20	160	40	10	310	1	0.5
System 44	protein-free	R,R-HNK	40	120	80	10	310	1	0.5
System 45	protein-free	R,R-HNK	0	200	0	20	310	1	0.5
System 46	protein-free	R,R-HNK	20	160	40	20	310	1	0.5
System 47	protein-free	R,R-HNK	40	120	80	20	310	1	0.5
System 48	protein-free	R,R-HNK	0	200	0	40	310	1	0.5
System 49	protein-free	R,R-HNK	20	160	40	40	310	1	0.5
System 50	protein-free	R,R-HNK	40	120	80	40	310	1	0.5

† These simulations were performed in the NVT ensemble, after initial equilibration at 310 K to achieve the correct area per lipid. A flat-bottomed half-harmonic restraint (force constant of 1000 kJ/mol/nm^2) was used for TRKB systems to keep the inter-helical distance between the Gly443 Cα atoms below 0.45 nm during the simulations to prevent dissociation of the helices and to achieve proper local sampling (see details above). All other simulations were performed in the NpT ensemble.
