# Supplementary material for: Antidepressant drugs act by directly binding to TRKB neurotrophin receptors
Source: Cell. 2021 Mar 4;184(5):1299–1313.e19. doi: 10.1016/j.cell.2021.01.034 (PMC7938888; doi:10.1016/j.cell.2021.01.034)
Supplement: Table S2. Levels of fluoxetine (15 mg/kg in the drinking water for 21 days) in the prefrontal cortex of mice, related to Figures 5, 6, and S6. [file mmc2.pdf]

Table S2. Levels of fluoxetine (15mg/kg in the drinking water for 21 days) in the prefrontal cortex of mice.

Mouse ID	genotype	tissue (mg)	FLX ug/ml	ug/g wet tissue	ug/ml*	uM**
PC1260	wt	32.0	1.575	9.846	10.29	33.30
PC1262	wt	33.6	1.497	8.908	9.31	30.13
PC1431	wt	20.4	0.393	3.856	4.03	13.04
PC1451	wt	30.8	1.642	10.662	11.14	36.06
PC1452	wt	30.9	1.024	6.629	6.93	22.42
PC1428	het	29.5	1.368	9.273	9.69	31.36
PC1439	het	12.6	0.379	6.013	6.28	20.34
PC1458	het	27.0	2.739	20.292	21.21	68.63

*** brain density: 1.045g/ml; ** fluoxetine MW: 309g/mol.**
